# Sex differences in pharmacological treatment of heart failure: a meta-analysis of randomized trials

**DOI:** 10.1093/eurheartj/ehag311

**Published:** 2026-04-28

**Authors:** Marte F van der Bijl, Niels T B Scholte, Julie A E van Oortmerssen, Jeanine E Roeters van Lennep, Astrid Schut, Iris C D Westendorp, Robert M A van der Boon, Jasper J Brugts, Isabella Kardys, Yolande Appelman, Hester M den Ruijter, Sanne A E Peters, Eric Boersma

**Affiliations:** Department of Cardiology, Cardiovascular Institute, Erasmus MC, University Medical Center, Doctor Molewaterplein 40, Rotterdam 3015 GD, The Netherlands; Department of Cardiology, Cardiovascular Institute, Erasmus MC, University Medical Center, Doctor Molewaterplein 40, Rotterdam 3015 GD, The Netherlands; Department of Epidemiology, Erasmus MC, University Medical Center, Rotterdam, The Netherlands; Department of Internal Medicine, Cardiovascular Institute, Erasmus MC, University Medical Center, Rotterdam, The Netherlands; Dutch Network for Cardiovascular Research (WCN), Utrecht, The Netherlands; Dutch Network for Cardiovascular Research (WCN), Utrecht, The Netherlands; Department of Cardiology, Red Cross Hospital, Beverwijk, The Netherlands; Department of Cardiology, Cardiovascular Institute, Erasmus MC, University Medical Center, Doctor Molewaterplein 40, Rotterdam 3015 GD, The Netherlands; Department of Cardiology, Cardiovascular Institute, Erasmus MC, University Medical Center, Doctor Molewaterplein 40, Rotterdam 3015 GD, The Netherlands; Department of Cardiology, Cardiovascular Institute, Erasmus MC, University Medical Center, Doctor Molewaterplein 40, Rotterdam 3015 GD, The Netherlands; Department of Cardiology, Amsterdam University Medical Center, Amsterdam, The Netherlands; Laboratory for Experimental Cardiology, Department of Cardiology, University Medical Center Utrecht, Utrecht University, Utrecht, The Netherlands; Julius Center for Health Sciences and Primary Care, University Medical Center Utrecht, Utrecht University, Utrecht, The Netherlands; Department of Cardiology, Cardiovascular Institute, Erasmus MC, University Medical Center, Doctor Molewaterplein 40, Rotterdam 3015 GD, The Netherlands

**Keywords:** Heart failure, Sex differences, Clinical trials as topic

## Abstract

**Background and Aims:**

Women remain underrepresented in heart failure (HF) trials, raising concerns about potential undetected sex-specific differences in treatment efficacy. This study assesses sex differences in the primary efficacy endpoint of HF treatments and examines whether the proportion of women enrolled in a trial influences (variations in) treatment effect estimates.

**Methods:**

A systematic search was conducted up to 21 March 2025, including randomized controlled trials (RCTs) (≥100 patients) evaluating pharmacological HF treatments vs. placebo or usual care, with clinical events as the primary outcome. Sex differences in the primary outcome were assessed using a random-effects meta-analysis of the reported relative effect measures (REM) and pooled estimates. For key clinical outcomes, meta-regression analyses were performed to examine the association between the proportion of women enrolled and sex differences in REMs, as well as the overall REMs without separating sex.

**Results:**

Of 5749 screened publications, 139 RCTs met inclusion criteria, with 292 027 patients (28.1% women). Based on 78 RCTs that reported sex-stratified treatment effects, pooled analysis showed no difference in treatment efficacy between women and men (delta ln[REM] 0.00; 95% confidence interval (CI) −0.04 to 0.03; *P* = .85; I^2^ = 4.1%). Meta-regression found no association between the proportion of women and sex differences in REM (78 RCTs, *P* = .25), overall efficacy (139 RCTs, *P* = .24), or other clinical outcomes.

**Conclusions:**

These findings suggest that pharmacological efficacy in HF does not differ by sex and that historical female underrepresentation in trials is unlikely to have masked important sex differences. Nonetheless, improving sex balance in HF trials remains essential for societal and ethical reasons.

## Graphical Abstract

**Graphical Abstract:**
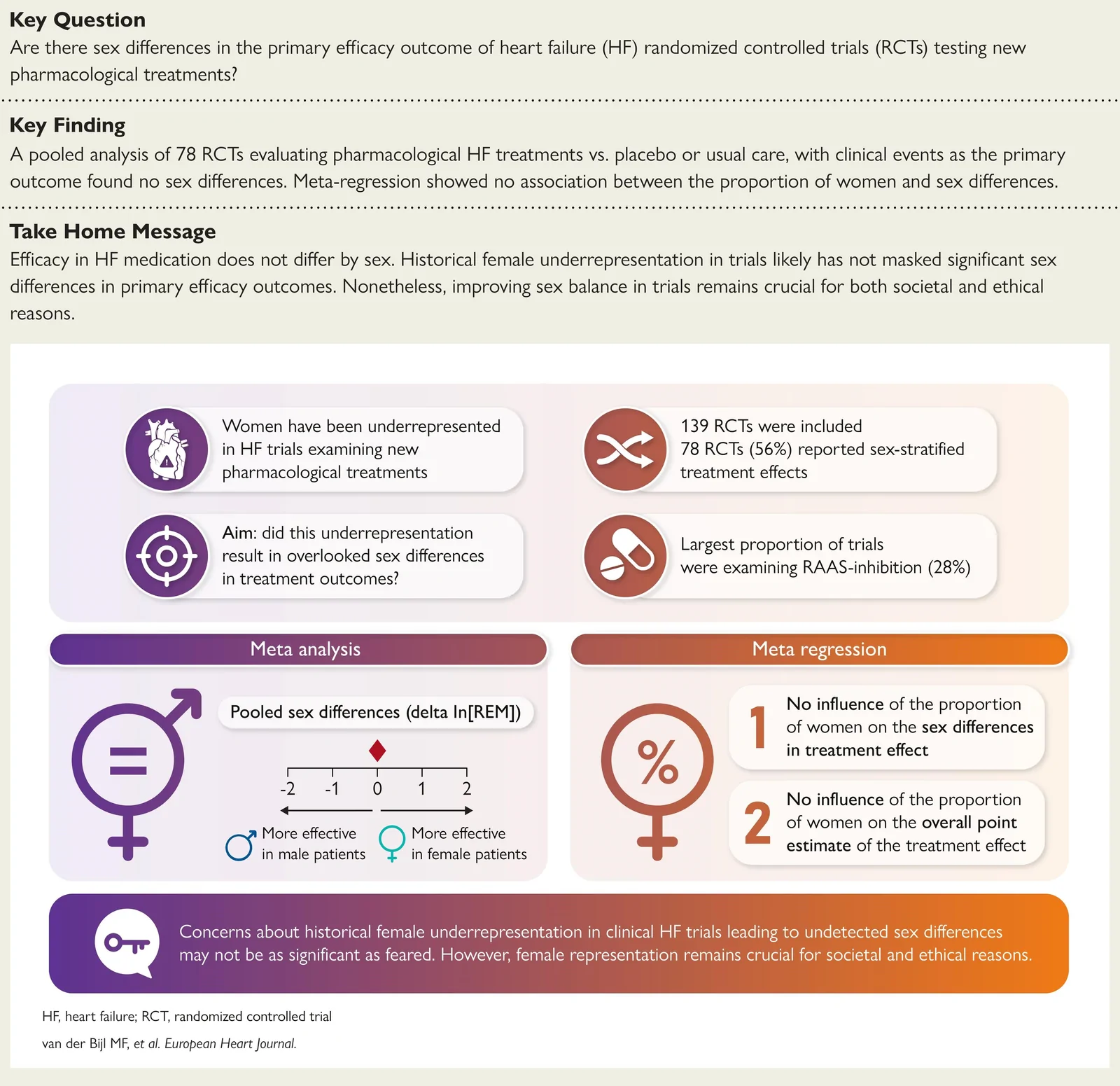

**See the editorial comment for this article ‘Do women and men have equal benefits from heart failure medications?’, by C. Linde, https://doi.org/10.1093/eurheartj/ehag559.**

## Introduction

Heart failure (HF) is a highly prevalent condition, affecting approximately 63 million people worldwide.^1^ Over half of the HF population are women,^2^ with well-established sex differences in the prevalence of various types of HF. Women are more likely to have HF with preserved ejection fraction (HFpEF), whereas HF with reduced ejection fraction (HFrEF) and HF with mildly reduced ejection fraction (HFmrEF) are more common in males.^3^ Additionally, women with HF generally exhibit better overall prognosis than men, irrespective of HF subtype.^4,5^ By contrast, women often report poorer quality of life and greater functional impairment compared with men.^6^ Moreover, in women, there is a higher incidence of comorbidities at time of diagnosis such as renal impairment and hypertension.^7^ Recent evidence also indicates that women, when adjusted for baseline differences, are less likely than men to receive guideline-directed medical therapy and have fewer annual cardiology follow-up visits.^8^ Despite these sex differences, along with general physiological variations between sexes that may affect pharmacodynamics and kinetics,^9^ current global HF guidelines do not recommend different (pharmacotherapeutic) treatment strategies for female and male patients.^10^

Relative to the population prevalence, women remain largely underrepresented in cardiovascular randomized controlled trials (RCTs) including HF.^11,12^ This disparity, also highlighted by the Lancet Women and Cardiovascular Disease Commission,^13^ might lead to uncertainty regarding the efficacy outcomes of HF therapies in women, especially in view of sex differences in aetiology and prognosis. Moreover, reporting on sex-specific safety data is rare,^14^ whereas the available data suggest that women in general report more adverse drug-related reactions.^15^ It is noteworthy that RCTs are typically not designed with adequate power to detect differences in treatment effects within a specific subgroup, a reason why subgroup results should be interpreted with caution.^16^ Meta-research offers a robust approach to address this limitation by aggregating data across studies, thereby enhancing the statistical power to evaluate sex-specific outcomes.^17^

In the domain of HF therapy, meta-analyses of pooled individual patient data have found consistent benefits of mineralocorticoid receptor antagonists, angiotensin-converting enzyme inhibitors, and beta-blockers for both female and male patients.^18,19^ Additionally, a meta-analysis regarding sex differences in the efficacy of guideline-directed medical therapy showed that guideline-directed medical therapy is as effective in female as in male patients.^20^ Nevertheless, the limited female representation in RCTs has remained a consistent concern, with ongoing debate about whether this long-standing underrepresentation contributes to undetected sex-related differences in treatment outcomes.^21,22^ A critical unresolved question is whether increasing female representation in RCTs would uncover meaningful sex-specific variations in therapeutic outcomes. Moreover, a comprehensive analysis including all RCTs on pharmacological therapies for HF, regardless of their inclusion in current guidelines, is lacking.

The aim of this meta-analysis and meta-regression is to evaluate sex differences in the efficacy of all pharmacological treatments for HF tested in RCTs and to investigate whether these differences are influenced by the proportion of women participating in the trials. Additionally, meta-regression is performed to examine the effect of the proportion of women in trials on other key clinical outcomes.

## Methods

### Protocol and registration

We performed a systematic review, meta-analysis, and meta-regression of RCTs and complied with the reporting standards of the Preferred Reporting Items for Systematic Reviews and Meta-Analyses (PRISMA) guideline.^23^ This review is prospectively registered in the International Prospective Register of Systematic Reviews (PROSPERO) under the number CRD42024522236.

### Terminology

Sex was defined as biological sex, as reported in the included RCTs, reflecting physiological and anatomical characteristics.^24^ However, we acknowledge that gender may also influence participation in trials and cardiovascular health more broadly. Accordingly, the terms ‘woman/women’ and ‘man/men’ are used throughout the manuscript to refer to patients participating in the trials. While we recognize the limitations of a binary classification, we report these data as documented in the original trial sources.

### Search strategy and selection criteria

In collaboration with an information specialist from the medical library of Erasmus MC, a literature search was carried out using Medline Ovid and Embase. The search included studies reported until 21 March 2025, with no specified starting date. We searched for RCTs using the following keywords: ‘Heart failure’, ‘Drug therapy’, and ‘Randomized controlled trials’. The full search strategy for Medline Ovid is provided in Supplementary Appendix 1. Only published peer-reviewed original articles in the English language were included in our study. Studies were considered eligible for inclusion if they had a RCT design assessing a clinical outcome comparing any pharmacological treatment for HF with placebo or standard of care in HF patients aged 18 years or older. To minimize the likelihood of small-study effects, all trials were required to have at least 100 participants.^25^ Two reviewers (M.B. and N.S.) independently performed title and/or abstract screening to identify articles who potentially met the eligibility criteria. Covidence systematic software (Veritas Health Innovation, Melbourne, Australia; available at www.covidence.org) was used to screen the articles. Afterwards article selection via full-text was performed. In case of disagreement regarding eligibility, consensus was reached in consultation with the supervising author (E.B.).

### Data extraction and quality assessment

The following predefined information was extracted from the main articles: trial characteristics (authorship, publication year, and region of origin), patient characteristics (age, sex, left ventricular ejection fraction, and NYHA class), details regarding the intervention and comparison group (type of medication and use of placebo), any event-driven outcomes (relative association measures and absolute numbers). If trials reported relative effect measures (REMs), then these were used in our analyses, and if trials only reported absolute numbers, odds ratios (ORs) were calculated. When sex-stratified data for the primary outcome were not available in the main publication, we searched for additional sex-specific analyses published separately and retrieved these data where possible. For all included RCTs, inclusion and exclusion criteria were reviewed, using either the primary publication or related rationale paper, to identify any criteria that explicitly differed between female and male patients.

The data were extracted from the articles by M.B. and N.S. independently. Risk of bias assessment was carried out for each article using the Cochrane risk of bias (RoB2) tool for RCTs. For each article, two independent reviewers (J.O. and M.B.) performed risk of bias analysis.

### Data synthesis and analysis

Trial characteristics are presented as means with ranges. Patient characteristics are presented as weighted means with ranges for continues variables and mean percentage with range for categorical variables.

Within the meta-analysis, the sex-stratified ln-transformed REM of the primary efficacy outcome, as selected by the trial, is used. Sex differences for the primary efficacy outcome, defined as delta ln(REM), were calculated by subtracting the ln-transformed REM for the primary outcome in female from that in male patients. The standard error (SE) of delta ln(REM) was calculated as the square root of the sum of the squared SEs of ln(REM) for women and men. Simple random-effects methods, using the DerSimonian and Laird procedure, were applied to estimate the pooled delta ln(REM), which is reported with the corresponding 95% CI. Pharmacological interventions were further classified according to the type of treatment investigated, reflecting the four pillars of chronic heart failure therapy: beta-blockers, renin–angiotensin–aldosterone system (RAAS) inhibitors, mineralocorticoid receptor antagonists, and sodium–glucose cotransporter 2 (SGLT2) inhibitors, as well as therapies used in acute HF, including diuretics, vasodilators, and inotropic agents.^26^ Interventions that did not fit into these categories were classified as ‘other’. A multi-level random-effects meta-analysis was performed to assess subgroup differences between medication types. Heterogeneity was assessed using the I^2^-statistic and classified as not important (I^2^: ≤25%), moderate, (I^2^: 26%–50%), substantial (I^2^: 51%–75%), and considerable (I^2^: >75%).^27^ Funnel plots were generated and assessed visually. A multi-level random-effects meta-analysis was conducted, stratified by placebo-controlled trials, trials involving patients with chronic HF, and trials with a low risk of bias. Given the higher prevalence of HFpEF in women, sensitivity analyses were conducted on HFpEF trial data only, stratified according to the commonly used ejection fraction cutoffs at the time the studies were conducted and in accordance with the prevailing guidelines.^28^

A meta-regression was conducted to examine the relationship between the proportion of women in each trial and sex differences in the relative treatment effect for the primary efficacy endpoint (delta ln[REM]), as well as the overall relative treatment effect without separating sex (ln[REM_overall_]). Additionally, meta-regression was performed to examine the relationship between the percentage of female participants and the overall relative treatment effect without separating sex (ln[REM_overall_]) for all-cause mortality, cardiovascular mortality, and HF hospitalizations. Over the years, the definition of the different subtypes of HF has evolved.^28^ To address this, meta-regression was performed on the mean left ventricular ejection fraction of each study and sex differences in the primary efficacy endpoint (delta ln[REM]). For all meta-regression analyses, a simple random-effects meta-regression using restricted maximum likelihood was used.

All analyses and visualizations were performed using the ‘meta’ and ‘metafor’ packages in R Studio (version 4.2.2). A two-sided *P*-value of ≤.05 was considered as statistically significant.

## Results

### Trial and patient characteristics

After duplicate removal, the literature search yielded 5749 articles, of which 211 were eligible for full-text review. An overview of the excluded studies from full-text review and reasons can be found in Supplementary data online, *Table S1*. From the full-text review, 139 RCTs met all eligibility criteria and were included in the analysis (*Figure 1*).^29–167^ Within the included trials, a total of 292 027 patients were enrolled, of whom 81 931 (28.1%) were women (*Table 1* and Supplementary data online, *Table S2*). The percentage of women enrolled in these trials ranged from 1% to 60%. Among the included trials, six articles had multiple treatment arms.^52,57,86,89,119,143^ The included trials assessed a variety of primary efficacy endpoints and pharmacological interventions (see Supplementary data online, *Table S2*). Nineteen RCTs (13.7% of all included RCTs) were judged to have some concerns regarding the risk of bias, mainly in the domain assessing the bias in selection of the reported results. All other were judged as low risk of bias (see Supplementary data online, *Figures S1* and *S2*). Of these trials, 52.5% included sex-specific inclusion or exclusion criteria, though in nearly all cases this was limited to the exclusion of pregnant or lactating women, with exemptions typically made for women of reproductive age using adequate contraception. Only eight additional trials used an upper age limit, which can disproportionately exclude female HF patients (see Supplementary data online, *Table S3*).

**Figure 1:**
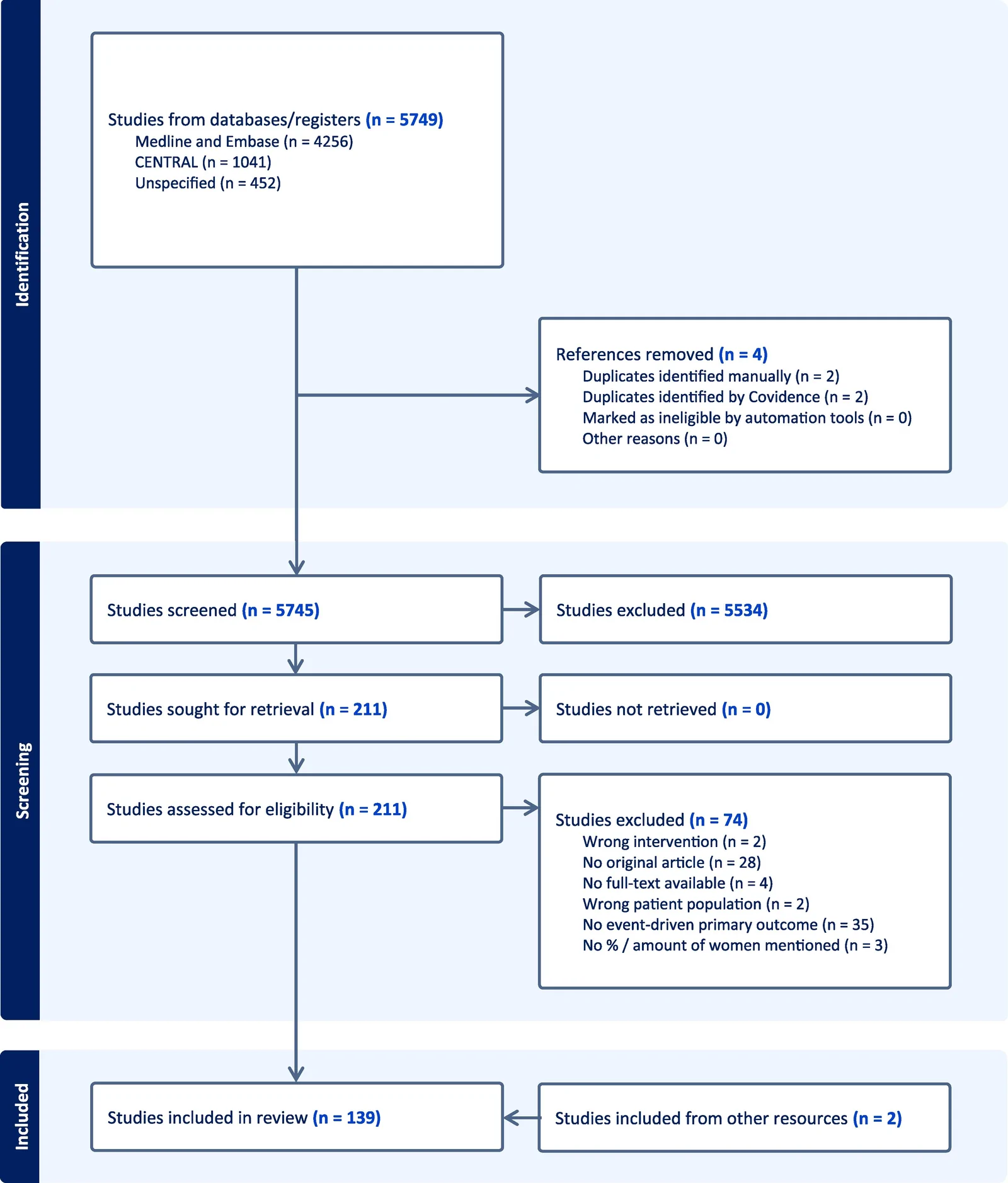
PRISMA flowchart

**Table 1:** Summary of trial and patient characteristics

Number of studies	139
Total number of participants	292 027
Number of participants, mean [range]	2101 [100–14703]
Age (years), mean [range]	69 [38–78]
Sex	
Women, *n* (%)	81 931 (28.1)
Women per study, mean % [range]	29.3 [1.0–72.8]
Men, *n* (%)	210 096 (71.9)
Men per study, mean % [range]	70.6 [27.2–98.9]
LVEF (%)^a^, mean [range]	33 [17–65]
NYHA class, mean % [range]	
I/II	56.5 [0–100]
III/IV	52.3 [0–100]
Ischaemic aetiology, mean % [range]	57.6 [0–100]

LVEF, left ventricular ejection fraction; NYHA, New York Heart Association.

^a^In two articles, only medians are available, from which it was not possible to estimate the mean. In these cases, medians were used.

### Sex differences in efficacy of pharmacological treatment of heart failure

Sex-stratified data on the REM for the primary outcome were available in 74 RCTs, and four additional studies were identified through secondary analyses or meta-analyses specifically examining sex differences^19,168^ (see Supplementary data online, *Table S4*). These 78 RCTs (56.1% of included trials) included two trials with multiple treatment arms, resulting in a total of 80 treatment comparisons. All these trials included 237 655 patients (81.4% of all included patients), 27.7% of whom were women.

The largest proportion of trials (28.2%) were classified as investigating RAAS inhibition (see Supplementary data online, *Table S4*). Most trials (60.3%) employed composite endpoints, comprising various component outcomes. The majority of trials were focusing on the treatment of chronic HF patients (75.6% of trials) and were placebo-controlled (74.4% of trials).

The pooled analysis showed no sex differences in the primary outcome between female and male patients for pharmacological HF therapy (delta ln[REM]: 0.00, 95% CI −0.04 to 0.03; *P* = .85; heterogeneity I^2^: 4.1%) nor within any specific pharmacological category. The results are visualized in *Figure 2*. Funnel plot showed no evidence for publication bias (see Supplementary data online, *Figure S3*). Sensitivity analyses that were restricted to trials with a low risk of bias (76 trials), placebo-controlled trials (58 trials), those enrolling patients with chronic HF (60 trials), and HFpEF (9 trials) yielded results consistent with the overall meta-analysis findings. With regard to the HFpEF trials, it should be noted that preserved ejection fraction was defined as 40%–50% in six trials, >50% in one trial, and by only a broad reference to ‘HFpEF’ in one trial.

**Figure 2:**
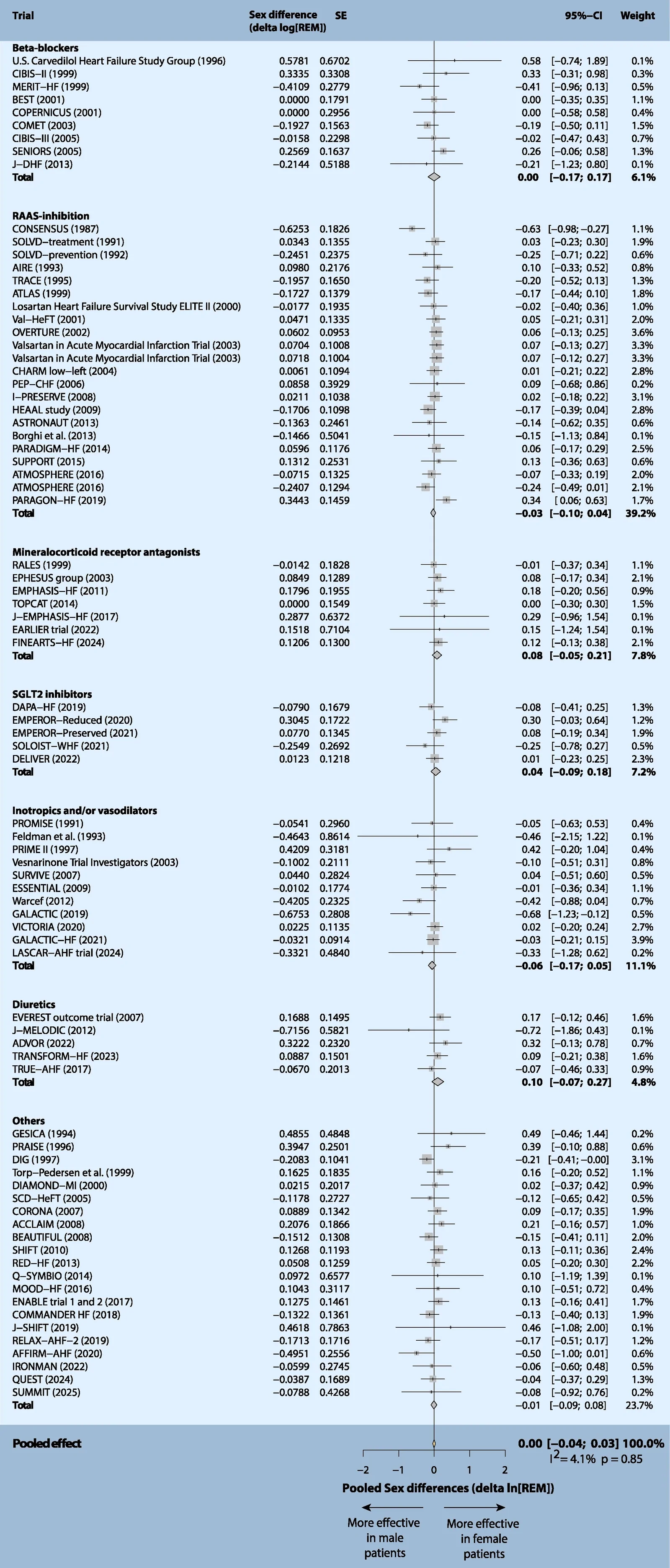
Forest plot of meta-analysis on pooled sex differences (delta ln[REM]). Trials that investigated multiple treatments appear once per treatment

### Meta-regression analyses

In meta-regression analyses, the proportion of women in a trial was not related to the difference in REM between female and male patients on the primary endpoint (78 trials, *P* = .25), neither with the overall point estimate in women and men combined (139 trials, *P* = .24) (*Figure 3*), a sensitivity analysis stratified by effect measure yielded consistent results. Furthermore, no association was observed between the proportion of women in a trial and the overall REM of all-cause mortality (127 trials, *P* = .45), cardiovascular mortality (83 trials, *P* = .29), and HF hospitalizations (79 trials, *P* = .40) (*Figure 4*).

**Figure 3:**
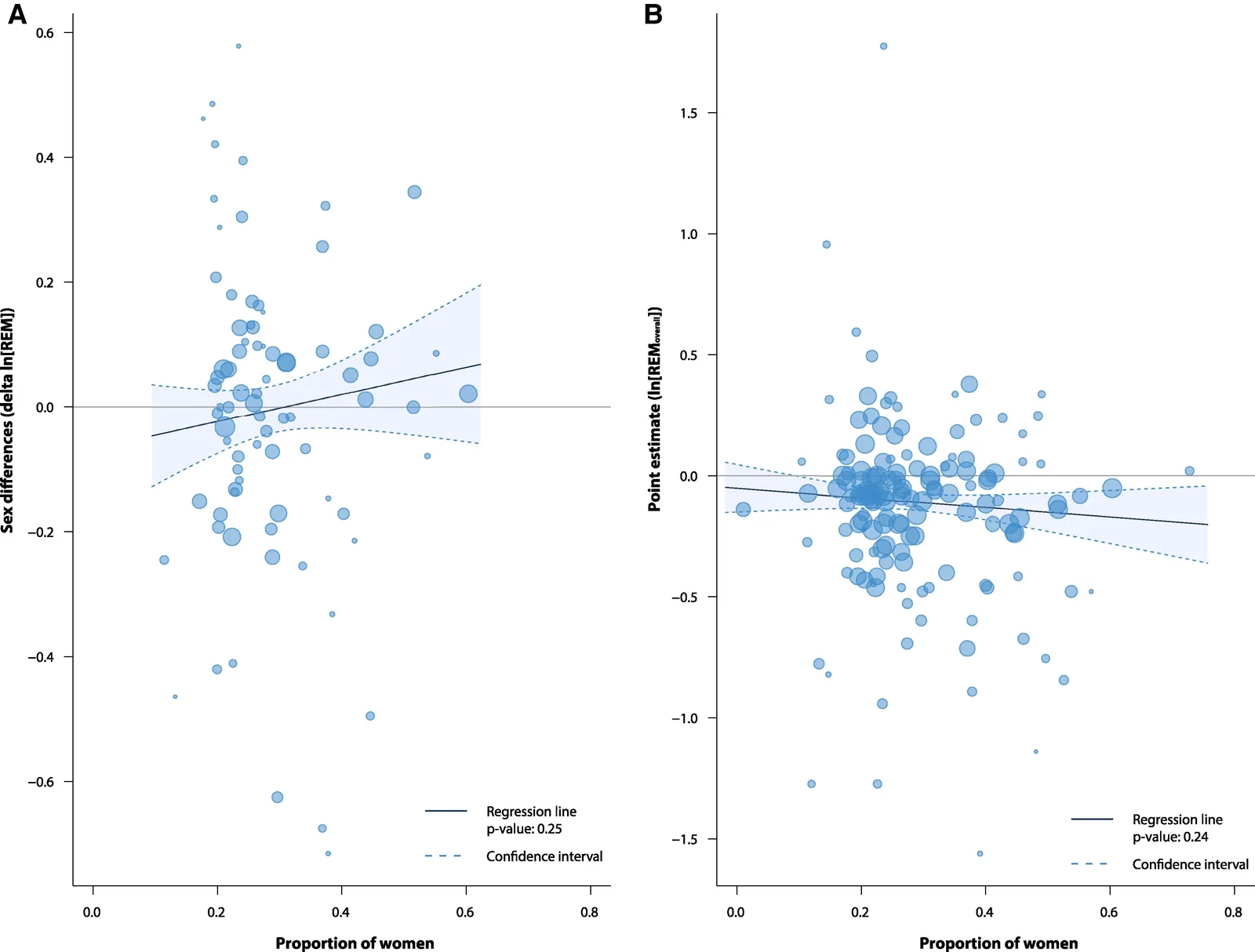
Bubble plots of meta-regression on primary efficacy endpoints of the trial. (*A*) Sex differences (delta ln[REM]) by proportion of women in the trial. (*B*) Point estimate (ln[REM_overall_]) by the proportion of women in the trial

**Figure 4:**
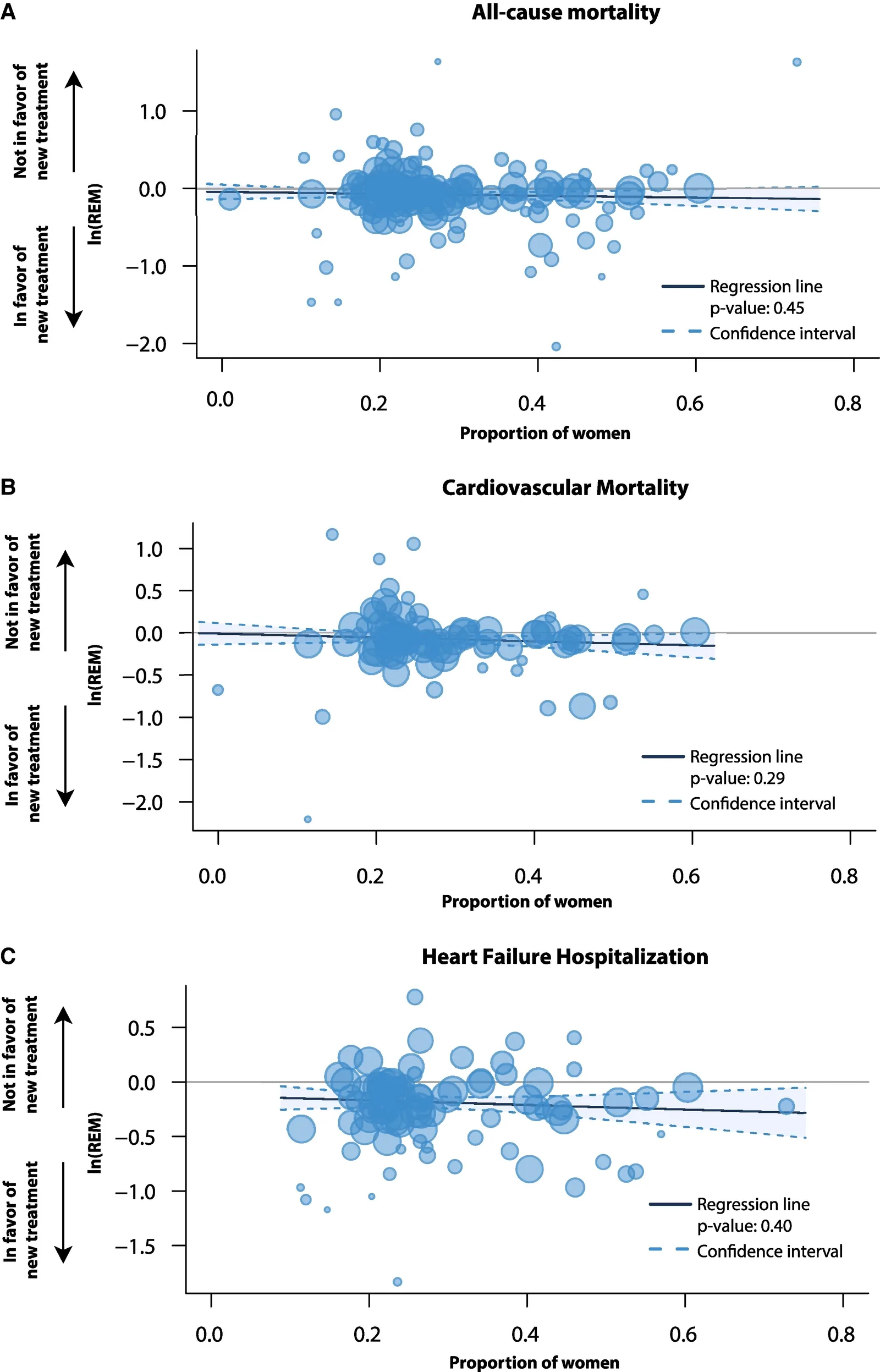
Meta-regression results of the proportion of women in the trial on the REM of specific outcomes: (A) All-cause mortality. (B) Cardiovascular mortality. (C) Heart failure hospitalization

Meta-regression analyses were additionally performed incorporating absolute numbers of women, which served as a proxy for statistical power, based on the rationale that a higher female enrolment would be associated with a greater number of events among women. These analyses revealed no significant associations between the absolute number of women included and sex-specific differences, effect size estimates, or other key clinical outcomes. The figures are added to the supplementary documents (see Supplementary data online, *Figures S4* and *S5*).

We also investigate the relation between left ventricular ejection fraction (used to distinguish between HFpEF, HFmrEF, and HFrEF) and sex differences in primary efficacy endpoint, which appeared absent (71 trials, *P* = .70) (see Supplementary data online, *Figure S6*).

The use of pharmacological agents can be associated with serious adverse events (SAEs). Therefore, we conducted an exploratory analysis to examine whether sex differences in SAEs were also apparent in the trials included in our meta-analysis, defined as the occurrence of at least one SAE per patient. Notably, only 42 RCTs (30.2% of the included trials) provided standardized safety data, defined as the number of patients with at least one reported SAE. In many trials, safety reporting was inconsistent: some reported only the total number of SAEs, while others used alternative definitions, such as adverse events leading to treatment withdrawal. Unfortunately, none of the 42 trials reported sex-stratified data on SAEs. Still, meta-regression was possible, which showed no association between the proportion of female participants in a trial and the differential occurrence of adverse events (see Supplementary data online, *Figure S7*).

## Discussion

This meta-analysis, including sex-stratified efficacy information from 78 trials with a total of 237 655 patients (81.4% of all included patients and 27.7% women) with HF, demonstrates no differences in efficacy of pharmacological therapy between female and male patients. Meta-regression showed no association between the proportion of women in the trials and potential sex differences in treatment efficacy. Similarly, meta-regression of all 139 included trials indicated that the proportion of women enrolled does not influence the point estimate of the primary outcome or the relative estimates of key clinical HF outcomes, including all-cause mortality, cardiovascular mortality, and HF hospitalization (*Structured Graphical Abstract*).

Our findings align with a prior smaller meta-analysis assessing guideline-directed medical therapy in HF, which reported no relevant sex-based differences in treatment efficacy in a sample of 84 818 patients (27% women).^20^ These results are also consistent with analyses of individual drug classes, including a pooled analysis of three RCTs on mineralocorticoid receptor antagonists with 6167 patients (31% women),^18^ as well as a meta-analysis of the 12 largest RCTs evaluating angiotensin-converting enzyme inhibitors and beta-blockers including 10 213 patients (23% women).^19^ Our study extends beyond guideline-directed medical therapy and single drug classes by encompassing all tested HF therapies, regardless of the trial outcomes or their inclusion in the guideline.

An observational study by Santema *et al*. suggested that women with HFrEF may achieve optimal efficacy at approximately half the guideline-recommended doses, with no additional benefit at higher doses.^169^ Due to its *post hoc* design, this study by Santema *et al*. was unable to assess potential dose-related adverse effects of serum drug levels. Given the design of current RCTs, which test a fixed dosage, these aspects cannot be reliably evaluated within the existing trial framework nor in the present meta-analysis of these trials. Future, well-powered Phase II dose-finding studies are needed to explore potential sex differences in optimal dosing.

Our findings provide reassurance for clinical practice and, importantly, suggest that the historical female underrepresentation in HF trials is unlikely to have resulted in missed sex-based differences in the efficacy of pharmacological therapy. By ‘adequate representation’, we refer to enrolment that reflects the sex distribution of the target HF population for the specific phenotype and clinical context under study, rather than a fixed numerical quota. Because HF phenotypes vary in prevalence by sex, representation should be interpreted in light of real-world epidemiology and the trial’s scientific objectives. Such alignment supports both the ethical principle of equitable participation and the methodological goal of ensuring generalizability of treatment effects.^170^ The participant-to-prevalence ratio (PPR) may serve as a useful benchmark in this regard,^171^ although obtaining reliable phenotype- and context-specific PPR estimates remains challenging.

The primary aim of this work was to examine the methodological consequences of female underrepresentation in HF trials. We acknowledge, however, that issues of underrepresentation in HF research extend beyond sex and include other populations, such as older adults, racial and ethnic minorities, and individuals with lower socioeconomic status.^172^ The narrow CIs and low heterogeneity further support the absence of a systematic sex difference. Hence, given that (Phase III) RCTs are primarily designed to assess treatment efficacy, our findings do not support the need for a fixed men-to-women enrolment ratio or women-only trials when evaluating efficacy. From an ethical and societal standpoint, adequate representation of women in clinical HF trials remains essential. Such representation ensures an equitable distribution of the risks and benefits of trial participation and helps maintain trust in medical research, particularly when patients do not see themselves reflected in study populations.^173^ Our meta-regression provides reassurance that the available evidence regarding treatment efficacy is applicable to both female and male HF patients. Nevertheless, we emphasize that enrolling a representative population of women remains critically important, and meta-regression should not be viewed by investigators as a substitute for adequate trial representation.

Furthermore, because sex-specific exclusion criteria in the included trials were limited to pregnancy and lactation, our findings are generalizable to the broader female population with HF. However, as previously discussed,^174^ despite the small number of affected patients, improved registries are needed to assess the long-term risks of nontreatment during pregnancy and lactation. Additionally, pregnancy and lactation should be considered separately, as conflating them may result in unnecessary exclusion from research. Finally, more indirect inclusion and exclusion criteria, such as age restrictions that may disproportionately limit female participation, were reported in 10% of the trials included in the meta-analysis. Nevertheless, the literature indicates that current HF trial populations are not fully representative of real-world populations, with women with HF being underrepresented.^175^ It remains unclear whether this underrepresentation is driven primarily by the cumulative effects of restrictive eligibility criteria or by lower willingness or opportunity to participate. Further research is needed to disentangle these contributing factors.

The lack of sex-stratified safety data and inconsistent reporting of serious adverse events highlight a critical gap in current research. To address this, the focus must extend beyond simply increasing female participation in Phase III RCTs to include sex-stratified reporting and standardized documentation of serious adverse events. This would allow for a more thorough assessment of potential sex differences in safety outcomes. Additionally, Phase IV pharmacovigilance studies could provide further insights into sex-specific side-effect profiles—which are known to be different between female and male patients^176^—in the real-world setting. Finally, beyond the scope of clinical trials, the integration of real-world evidence into pharmacogenomics may enable investigation of the contribution of sex chromosome variation to drug-specific effects, as exemplified by recent sex-specific Mendelian randomization analysis in lipid-lowering therapies.^177^

A few limitations should be acknowledged. First, this meta-analysis focuses exclusively on pharmacological therapies. In the management of advanced HF, device-based therapies, such as implantable cardioverter–defibrillators and cardiac resynchronization therapy, also play an important role.^178^ Women are substantially underrepresented in device trials as well,^179^ particularly in the cardiovascular field, representing an additional area in which further investigation may be informative. Second, this meta-analysis is based on aggregated (trial-level) data rather than individual patient-level data, which limits the precision of effect estimates and may introduce ecological bias. In particular, trial-level analyses cannot fully account for within-trial sex-by-treatment interactions and may therefore obscure true individual-level sex-specific treatment effects that could be identified using individual patient-level data. Third, the meta-analysis pools trials across different HF phenotypes, clinical contexts, and therapeutic classes, thereby introducing considerable clinical heterogeneity. However, sensitivity and stratified analyses across HF phenotypes and therapeutic domains yielded consistent results and supported the same overall conclusion. Moreover, this broad analytical approach is necessary to assess whether systematic biases in treatment effects favour either female or male patients. Finally, a limitation of this study is the heterogeneity in outcomes and effect measures reported across the included RCTs, including hazard ratios, risk ratios, and ORs. Although we used the difference in log REMs to enable comparison of sex-specific treatment effects on a common scale, this approach cannot fully resolve all challenges associated with differing outcome metrics. While sensitivity analyses stratified by effect measure yielded consistent results, individual patient-level data meta-analyses would provide a more robust framework to harmonize outcome definitions and effect measures and to allow more refined assessment of sex-specific treatment effects.

In this comprehensive systematic review and meta-analysis of RCTs evaluating pharmacological therapy for HF, we found no evidence of sex-based differences in treatment efficacy. Meta-regression analyses further demonstrated that the proportion of women enrolled in a trial did not influence the primary outcome or other key clinical endpoints, including all-cause mortality, cardiovascular mortality, and HF hospitalization. Similarly, in an exploratory analysis of safety, using meta-regression analysis, no association was observed between the proportion of women and the occurrence of serious adverse events. These findings provide reassurance that the historical female underrepresentation in HF trials has not led to overlooked sex differences in treatment efficacy.

## Supplementary Material

ehag311_Supplementary_Data

## Declarations

### Data Availability

Data extracted from published articles and used in our analysis will be made available upon request via email to the corresponding author.
