# Supplementary material for: Sex differences in pharmacological treatment of heart failure: a meta-analysis of randomized trials
Source: Eur Heart J. 2026 Apr 28;47(38):5527–41. doi: 10.1093/eurheartj/ehag311 (PMC13651700; doi:10.1093/eurheartj/ehag311)
Supplement: Supplementary file 1 [file ehag311_supplementary_data.docx]

**Supplementary appendix**

**Table of Contents**

[Appendix 1 Search term used in MEDLINE search.	2](#_Toc219367852)

[Supplementary table S1 Articles excluded from full-text review (1-74)	3](#_Toc219367853)

[Supplementary table S2 Detailed overview of study and patient characteristics	5](#_Toc219367854)

[Supplementary table S3 Sex specific in and exclusion criteria	13](#_Toc219367855)

[Supplementary table S4 Sex-specific relative effect measures of the primary endpoint	17](#_Toc219367856)

[Supplementary figure S1 Risk of bias analysis per study.	20](#_Toc219367857)

[Supplementary figure S2 Risk of bias analysis - summary figure.	23](#_Toc219367858)

[Supplementary figure S3 Funnel plot.	23](#_Toc219367859)

[Supplementary figure S4A+B Bubble plots: absolute number of women	23](#_Toc219367860)

[Supplementary figure S5A-C Bubble plots: absolute number of women	24](#_Toc219367861)

[Supplementary figure S6 Bubble plot: left ventricular ejection fraction	24](#_Toc219367862)

[Supplementary figure S7 Bubble plot: serious adverse events	25](#_Toc219367863)

[Supplementary references	25](#_Toc219367864)

## Appendix 1 Search term used in MEDLINE search.

(Heart Failure / OR Heart Failure, Diastolic / OR Heart Failure, Systolic / OR (((heart* OR cardiac) ADJ3 (failure* OR insufficien* OR overload*)) OR ((diastolic OR systolic) ADJ3 dysfunction*)).ab,ti,kw.) AND (Drug Therapy / OR exp heart failure/dt OR ((drug* ADJ3 therap*) OR medication* OR pharmacotherap* OR (pharmac* ADJ3 therap*)).ab,ti,kw.) AND (Randomized Controlled Trial / OR ((random* ADJ3 (stud* OR trial))).ab,ti,kw.) AND (((0## OR 1## OR 2## OR 3## OR 4## OR 5## OR 6## OR 7## OR 8## OR 9## OR 1### OR 2### OR 3### OR 4### OR 5### OR 6### OR 7### OR 8### OR 9###) ADJ6 (patients OR patient-record* OR patient-file* OR subjects OR individuals OR cases OR persons OR men OR women OR males OR females OR participant* OR people OR children OR adolescent* OR boys OR girls OR teens OR teenagers OR infants OR newborns OR elderly OR survivor* OR specimen* OR sample* OR episode* OR isolate* OR pediatric OR paediatric OR adult* OR surgeries OR operations OR observations OR mother* OR father* OR neonate* OR volunt* OR recruit* OR infect* OR donor* OR presented OR treatments )) OR ((hundred* OR thousand* OR million*) ADJ6 (patients OR patient-record* OR patient-file* OR subjects OR individuals OR cases OR persons OR men OR women OR males OR females OR participant* OR people OR children OR adolescent* OR boys OR girls OR teens OR teenagers OR infants OR newborns OR elderly OR survivor* OR specimen* OR sample* OR episode* OR isolate* OR pediatric OR paediatric OR adult* OR surgeries OR operations OR observations OR mother* OR father* OR neonate* OR volunt* OR recruit* OR infect* OR donor* OR presented OR treatments )) OR ((n OR included OR recruited OR randomized OR randomized OR assigned OR a-total-of OR group-of OR groups-of OR cohort-of OR cohorts-of) ADJ (0## OR 1## OR 2## OR 3## OR 4## OR 5## OR 6## OR 7## OR 8## OR 9## OR 1### OR 2### OR 3### OR 4### OR 5### OR 6### OR 7### OR 8### OR 9###)) OR large*-cohort* OR population-based OR multi-register* OR multiregister* OR regist*-based OR multicent* OR multi-cent* OR national-stud* OR national-regist* OR N#1## OR N#2## OR N#3## OR N#4## OR N#5## OR N#6## OR N#7## OR N#8## OR N#9## OR N#1### OR N#2### OR N#3### OR N#4### OR N#5### OR N#6### OR N#7### OR N#8### OR N#9###).ab,ti. NOT (Systematic Review / OR Meta-Analysis / OR (systematic-review* OR meta-analys*).ti.) NOT (exp animals/ NOT humans/) AND english.la.

## Supplementary table S1 Articles excluded from full-text review (1-74)

| **Author and year of publication** | **Reason of exclusion** |
| --- | --- |
| Abraham 2010 | No original article |
| Ali 2024 | No full-text available |
| Altenberger 2014 | No event-driven primary outcome |
| Anand 2004 | No event-driven primary outcome |
| Anderson 2003 | No original article |
| The Xamoterol in Severe Heart Failure Study Group 1990 | No event-driven primary outcome |
| Australia/New Zealand Heart Failure Research Collaborative Group | No event-driven primary outcome |
| Bartunek 2017 | Wrong intervention |
| Bergler-Klein 1994 | No full-text available |
| Boccanelli 2009 | No event-driven primary outcome |
| Butler 2022 | No event-driven primary outcome |
| Chen 2013 | No event-driven primary outcome |
| Chung 2003 | No event-driven primary outcome |
| Cleland 2024 | No original article |
| Cohn 1991 | No proportion or amount of women mentioned |
| Cowley 1994 | No event-driven primary outcome |
| Cuffe 2002 | No event-driven primary outcome |
| Davies 1991 | No event-driven primary outcome |
| DiBianco 1989 | No event-driven primary outcome |
| Erdmann 2013 | No event-driven primary outcome |
| Feldman 2007 | No event-driven primary outcome |
| Ghose 1993 | No full-text available |
| Granger 2000 | No event-driven primary outcome |
| Granger 2003 | No original article |
| Greenberg 2016 | Wrong intervention |
| Holubarsch 2010 | No original article |
| Hori 2004 | No event-driven primary outcome |
| Jespersen 1993 | No original article |
| Jha 2022 | No proportion or amount of women mentioned |
| Just 1993 | No original article |
| Kjekshus 1989 | No original article |
| Kosiborod 2023 | No event-driven primary outcome |
| Kosiborod 2022 | No original article |
| Kubo 1992 | No event-driven primary outcome |
| Ledwidge 2023 | No event-driven primary outcome |
| Mann 2022 | No event-driven primary outcome |
| Margulies 2016 | No event-driven primary outcome |
| Massie 1996 | Wrong patient population |
| Massie 2010 | No event-driven primary outcome |
| McMurray 2003 | No original article |
| Mentz 2023 | No event-driven primary outcome |
| Mentz 2023 | No original article |
| Morisco 1993 | No original article |
| Mortensen 2013 | No original article |
| Mortensen 2013 | No original article |
| Mortensen 2013 | No original article |
| Nieminen 2008 | No event-driven primary outcome |
| Packer 2021 | No original article |
| Packer 1996 | No event-driven primary outcome |
| Packer 2015 | No original article |
| Pang 2017 | No event-driven primary outcome |
| Petrie 2020 | No original article |

**Supplementary table S1** *Continued*.

| **Author and year of publication** | **Reason of exclusion** |
| --- | --- |
| Pfeffer 2003 | No original article |
| Pratt 2004 | No original article |
| Remme 1994 | No event-driven primary outcome |
| Rouleau 2000 | No event-driven primary outcome |
| Suzuki 2014 | No original article |
| Swedberg 1988 | No original article |
| Szarek 2021 | No original article |
| Szarek 2021 | No original article |
| Taylor 2004 | No event-driven primary outcome |
| Teerlink 2009 | No event-driven primary outcome |
| Torp-Pedersen 1996 | No original article |
| Tsutsui 2019 | No event-driven primary outcome |
| Udelson 2007 | No event-driven primary outcome |
| Vizzardi 2014 | No proportion or amount of women mentioned |
| Voors 2019 | No event-driven primary outcome |
| Win 2012 | No original article |
| Yang 2017 | No event-driven primary outcome |
| Yang 2024 | No full-text available |
| Yusuf 1993 | No original article |
| Yusuf 1992 | No original article |
| Yusuf 2008 | Wrong patient population |
| Zeng 2003 | No event-driven primary outcome |
| Zhao 2021 | No original article |

## Supplementary table S2 Detailed overview of study and patient characteristics

| **First authors, year** | **Study abbreviation** | **Primary Endpoint** | **Treatment** | **Control treatment** | **N patients** | **Women (%)** | **LVEF** | **NYHA class I/II** | **NYHA class III/IV** | **Ischemic etiology** |
| --- | --- | --- | --- | --- | --- | --- | --- | --- | --- | --- |
| Acute Infarction Rampril Efficacy Study Investigators, 1993 | AIRE | ACM | Ramipril | Placebo | 1986 | 525 (26,4) | - | - | - | - |
| Ahmed et al., 2006 | DIG ancillary trial | Combined (HFH, HF mortality) | Digoxin | Placebo | 988 | 407 (41,2) | 56 | 78 | 22 | 56 |
| Angermann et al., 2016 | MOOD-HF | Combined (ACM, any hospitalization) | Escitalopram | Placebo | 372 | 91 (24,5) | 35 | 47 | 53 | 64 |
| Anker et al., 2021 | EMPEROR-Preserved | Combined (CV mortality, HFH) | Empaglifozin | Placebo | 5988 | 2676 (44,7) | 54 | 82 | 18 | 35 |
| Armstrong et al., 2020 | VICTORIA | Combined (CV mortality, first HFH) | Vericiguat | Placebo | 5050 | 1208 (23,9) | 29 | 59 | 41 | - |
| Asakura et al., 2022 | EARLIER trial | Combined (CV mortality, first cardiac rehospitalization) | Eplenerone | Placebo | 300 | 82 (27,3) | 31 | 42 | 57 | 36 |
| Bardy et al., 2005 | SCD-HeFT | ACM | Amiodarone | Placebo | 1692 | 398 (23,5) | 25 | - | - | - |
| Bhatt et al., 2021 | SOLOIST-WHF | Combined (CV mortality, HFH or urgent visits) | Sotagliflozin | Placebo | 1222 | 412 (33,7) | 35 | - | - | - |
| Borghi et al., 2012 | SMILE-4 | Combined (CV mortality, hospitalization) | Zofenopril | Ramipril | 716 | 172 (24) | 43 | - | - | - |
| Borghi et al., 2013 |  | CV mortality | Zofenopril | Ramipril | 175 | 55 (31,4) | 53 | 77 | 23 | - |
| Brown et al., 1995 | Fosinopril Heart Failure Study Group | ACM | Fosinopril | Placebo | 241 | 49 (20,3) | 25 | 91 | 9 | - |
| Califf et al., 1997 | FIRST | ACM | Epopresenol | Usual care | 471 | 113 (24) | - | 41 | 59 | 67 |
| Cheang et al., 2024 | QUEST | Combined (CV death, HFH) | QLQX (Chinese medicine) | Placebo | 3110 | 867 (27,9) | 32 | 53 | 47 | 75 |
| CIBIS-II investigators and committees, 1999 | CIBIS-II | ACM | Bisoprolol | Placebo | 2647 | 515 (19,5) | 28 | 0 | 100 | 50 |
| Cice et al., 2010 |  | ACM | Telmisartan | Placebo | 332 | 153 (46,1) | 30 | 34 | 67 | - |
| Cleland et al., 2004 | WASH | Combined (ACM, non-fatal MI and stroke) | I. Warfarin  II. Aspirin | I. and II. No anti-thrombotic agent | 279 | 72 (25,8) | - | 71 | 29 | 60 |
| Cleland et al., 2006 | PEP-CHF | Combined (ACM, HFH) | Perindopril | Placebo | 850 | 469 (55,2) | 65* | 76 | 24 | - |
| Clement et al., 2000 | CHIPS | Worsening HF | Captopril 50mg | Captopril 25 mg | 298 | 92 (30,9) | - | 50 | 50 | - |
| Cohn et al., 1998 | Vesnarinone Trial Investigators | ACM | Vesnarinone | Placebo | 2558 | 595 (23,3) | 21 | - | 99 | 59 |
| Cohn et al., 2001 | Val-HeFT | ACM | Valsartan | Placebo | 5010 | 1005 (20,1) | 27 | 62 | 38 | 57 |
| Cohn et al., 2003 | MOXCON | ACM | Moxonidine | Placebo | 1934 | 420 (21,7) | 26 | 42 | 58 | 63 |

**Supplementary table S2** *Continued*

| **First authors, year** | **Study abbreviation** | **Primary Endpoint** | **Treatment** | **Control treatment** | **N patients** | **Women (%)** | **LVEF** | **NYHA class I/II** | **NYHA class III/IV** | **Ischemic etiology** |
| --- | --- | --- | --- | --- | --- | --- | --- | --- | --- | --- |
| Cokkinos et al., 2006 | HELAS (in both I. ischemic and II. dilated cardiomyopathy patients) | Combined (ACM, non-fatal stroke, any embolism, MI, hospitalization, exacerbation of HF) | I. and II. Warfarin | I. Aspirin  II. Placebo | 197 | 30 (15,2) | 28 | - | - | 58 |
| CONSENSUS trial study group, 1987 | CONSENSUS | ACM | Enalapril | Placebo | 253 | 75 (29,6) | - | - | - | - |
| Cosín-Aguilar et al., 1999 |  | ACM | Captopril | Digoxin | 345 | 103 (29,9) | - | 70 | 30 | - |
| Cotter et al., 1998 |  | ACM | Isosorbidedinitrate | Furosemide | 104 | 50 (48,1) | 43 | - | - | 64 |
| Cowley et al., 1994 |  | ACM | Enoximone | Placebo | 151 | 29 (19,2) | - | 0 | 100 | 78 |
| Dargie et al., 2001 | CAPRICORN | Combined (ACM, CV hospitalization) | Carvedilol | Placebo | 1959 | 519 (26,5) | 33 | - | - | - |
| Doval et al., 1994 | GESICA | ACM | Amiodarone | Usual care | 516 | 99 (19,2) | 20 | 21 | 79 | - |
| Eichhorn et al., 2001 | BEST | ACM | Bucindolol | Placebo | 2708 | 593 (21,9) | 23 | 0 | 100 | 59 |
| EPOCH Study Group, 2002 | EPOCH | Combined (HF mortality, SCD, HFH) | Pimobedan | Placebo | 276 | 73 (26,4) | 33 | 66 | 34 | 34 |
| Feldman et al., 1993 |  | Combined (ACM, major CV morbidity) | Vesnarinone | Placebo | 477 | 63 (13,2) | 20 | 19 | 81 | 52 |
| Flather et al., 2005 | SENIORS | Combined (ACM, CV hospitalization) | Nebivolol | Placebo | 2128 | 785 (36,9) | 34 | 59 | 41 | - |
| Fonarow et al., 1992 | Hy-C | ACM | Hydralaxine | Captopril | 104 | 15 (14,4) | 20 | 0 | 100 | 59 |
| Fox et al., 2008 | BEAUTIFUL | Combined (CV death, MI, HFH) | Ivabradine | Placebo | 10917 | 1870 (17,1) | 32 | 76 | 23 | 100 |
| Gheorghiade et al., 2004 | ACTIV | Worsening HF | Tolvaptan | Placebo | 319 | 95 (29,8) | 24 | - | 95 | - |
| Gheorghiade et al., 2013 | ASTRONAUT | Combined (CV death, HFH) | Aliskiren | Placebo | 1615 | 368 (22,8) | 28 | 34 | 65 | 64 |
| Hampton et al., 1997 | PRIME II | ACM | Ibopamine | Placebo | 1906 | 374 (19,6) | 26 | - | - | 59 |
| Hoeper et al., 2024 | PASSION | Combined (ACM, HFH) | Tadalafil | Placebo | 125 | 91 (72,8) | - | 9 | 91 | - |
| Holubarch et al., 2008 | SPICE trial | Cardiac event | Crataegus extract | Placebo | 2681 | 435 (16,2) | 24 | 56 | 44 | - |
| Homma et al., 2012 | WARCEF | Combined (ACM, ischemic stroke, intracerebral hemorrhage) | Warfarin | Aspirin | 2305 | 465 (20,2) | 25 | 69 | 36 | 43 |
| Honda et al., 2024 | LASCAR-AHF | Combined (ACM, HFH) | Carperitide iv | Standard treatment | 247 | 95 (38,5) | 40 | 7 | 92 | 77 |

**Supplementary table S2** *Continued*

| **First authors, year** | **Study abbreviation** | **Primary endpoint** | **Treatment** | **Control treatment** | **N patients** | **Female sex (%)** | **LVEF** | **NYHA class I/II** | **NYHA class III/IV** | **Ischemic etiology** |
| --- | --- | --- | --- | --- | --- | --- | --- | --- | --- | --- |
| Jia et al., 2014 |  | Combined (ACM, MI, worsening HF) | Levosimendan | Placebo | 160 | 64 (40) | - | - | - | 29 |
| Karla et al., 2022 | IRONMAN | Combined (CV mortality, HF admission) | Ferric Derisomaltose IV | Usual care | 1137 | 300 (26,4) | 34 | 57 | 43 | 57 |
| Kawanami et al., 2025 |  | Worsening renal function | Dapagliflozin | Conventional therapy | 114 | 40 (35,1) | 33 | 33 | 67 | 23 |
| Kjekshus et al., 2007 | CORONA | Combined (CV mortality, nonfatal MI and stroke) | Rosuvastatin | Placebo | 5011 | 1180 (23,5) | 31 | 37 | 63 | 100 |
| Kjøller-Hansen et al., 2000 | APRES | Combined (cardiac mortality, nonfatal MI, HF, recurrent AP) | Ramipril | Placebo | 159 | 18 (11,3) | 42 | 47 | 53 | 100 |
| Kleber et al., 1992 |  | HF death | Captopril | Placebo | 170 | 42 (24,7) | - | - | - | - |
| Kober et al., 1995 | TRACE | ACM | Trandolapril | Placebo | 1749 | 501 (28,6) | - | 41 | - | - |
| Køber et al., 2000 | DIAMOND-MI | ACM | Dofetilide | Placebo | 1510 | 399 (26,4) | - | 65 | 36 | - |
| Køber et al., 2008 | ANDROMEDA | ACM or HFH | Dronedarone | Placebo | 627 | 155 (24,7) | - | 40 | 60 | 65 |
| Konstam et al., 2007 | EVEREST outcome trial | ACM | Tolvaptan | Placebo | 4133 | 1058 (25,6) | 28 | 0 | 100 | 66 |
| Konstam et al., 2009 | HEAAL study | Combined (ACM, HFH) | Losartan 150 mg | Losartan 50 mg | 3834 | 1143 (29,8) | 33 | 69 | 31 | 65 |
| Kozhuharov et al., 2019 | GALACTIC | Combined (ACM, HFH) | Early intensive vasodilatation | Usual care | 781 | 288 (36,9) | 37 | 0 | 100 | - |
| Lechat et al., 1994 | CIBIS | ACM | Bisoprolol | Placebo | 641 | 112 (17,5) | - | 0 | 100 | 55 |
| Levine et al., 2000 | MACH-1 | ACM | Mibefradil | Placebo | 2590 | 534 (20,6) | 25 | 26 | 75 | 68 |
| Massie et al., 2008 | I-PRESERVE | Combined (ACM, cardiac hospitalization) | Irbesartan | Placebo | 4128 | 2491 (60,3) | 60 | 23 | 80 | 25 |
| Massie et al., 2009 | WATCH | Combined (ACM, nonfatal MI or stroke) | I. and II. Warfarin  III. Clopidogrel | I. Clopidogrel  II and III. Aspirin | 1587 | 281 (17,7) | - | 44 | 56 | 73 |
| Masuyama et al., 2012 | J-MELODIC | Combined (CV death, HFH) | Azosemide | Furosemide | 320 | 121 (37,8) | 52 | 89 | 11 | 34 |
| Matsumori et al., 2003 | ARCH-J | Progression of CHF | Candesartan | Placebo | 292 | 66 (22,6) | 35 | 74 | 26 | 25 |
| McMurray et al., 2007 | VERITAS | Combined (ACM, worsening HF) | Tezosentan IV | Placebo | 1435 | 580 (40,4) | 29 | - | - | 68 |
| McMurray et al., 2014 | PARADIGM-HF | Combined (CV mortality or first HFH) | LCZ696 | Enalapril | 8399 | 1832 (21,8) | 30 | 75 | 25 | 60 |
| McMurray et al., 2016 | ATMOSPHERE | Combined (CV mortality or HFH) | I. Enalapril and Aliskren  II. Aliskiren | I. and II. Enalapril | 7016 | 1521 (21,7) | 28 | 63 | 37 | 56 |

**Supplementary table S2** *Continued*

| **First authors, year** | **Study abbreviation** | **Primary endpoint** | **Treatment** | **Control Treatment** | **N patients** | **Women (%)** | **LVEF** | **NYHA class I/II** | **NYHA class III/IV** | **Ischemic etiology** |
| --- | --- | --- | --- | --- | --- | --- | --- | --- | --- | --- |
| McMurray et al., 2019 | DAPA-HF | Worsening HF | Dapagliflozin | Placebo | 4744 | 1109 (23,4) | 31 | 68 | 32 | 56 |
| Mebazaa et al., 2007 | SURVIVE | ACM | Levosimendan IV | Dobutamine IV | 1327 | 371 (28) | 24 | - | - | 76 |
| Mentz et al., 2023 | TRANSFORM-HF | ACM | Torsemide | Furosemide | 2859 | 1055 (36,9) | - | - | - | 28 |
| MERIT-HF study group, 1999 | MERIT-HF | ACM | Metoprolol CR/XL | Placebo | 3991 | 898 (22,5) | 28 | 41 | 59 | 65 |
| Metra et al., 2009 | ESSENTIAL | CV mortality | Serelaxin IV | Placebo | 1854 | 372 (20,1) | 24 | 0 | 100 | 56 |
| Metra et al., 2019 | RELAX-AHF-2 | Combined (ACM, CV hospitalization) | Enoximone | Placebo | 6545 | 2637 (40,3) | 39 | 43 | 57 | 54 |
| Miller et al., 2008 |  | 30-day readmission | Nesiritide IV | Placebo | 101 | 35 (34,7) | - | 31 | 66 | - |
| Moiseyev et al., 2002 | RUSSLAN | Combined (hypotension of ischemia) | Levosimendan | Placebo | 504 | 244 (48,4) | - | - | - | 100 |
| Mortensen et al., 2014 | Q-SYMBIO | Combined (unplanned hospital stay for worsening HF, CV mortality, mechanical assist implantation, or urgent cardiac transplantation) | CoQ10 | Placebo | 420 | 115 (27,4) | 31 | 3 | 97 | 70 |
| Mullens et al., 2022 | ADVOR | Successful congestion | Acetazolamide IV | Placebo | 519 | 194 (37,4) | 43 | 13 | 87 | 45 |
| Muller et al., 2003 |  | Total number of hospitalizations | Torasemide | Furosemide | 237 | 135 (57) | - | - | - | - |
| Murray et al., 2001 |  | HFH | Torasemide | Furosemide | 234 | 123 (52,6) | - | - | - | - |
| Naser et al., 2023 |  | Combined (CV mortality or HFH) | Spironolactone | Eplerenone | 142 | 45 (31,7) | 34 | 57 | 43 | 35 |
| NETWORK investigators, 1998 | NETWORK | Combined (ACM, HFH, worsening HF) | Enalapril 2.5 mg | Enalapril 10 mg | 1022 | 362 (35,4) | - | 65 | 35 | 77 |
| Nicklas et al., 1991 |  | ACM | Amiodarone | Placebo | 101 | 15 (14,9) | 20 | 0 | 100 | 52 |
| O'Connor et al., 2003 | RITZ-4 | Combined (ACM, worsening HF, recurrent ischemia or MI) | Tezosentan IV | Placebo | 192 | 82 (42,7) | - | - | - | 100 |
| O'Connor et al., 2011 | ASCEND-HF | Combined (ACM, HFH) | Nesiritide IV | Placebo | 7007 | 2391 (34,1) | - | - | - | - |
| Packer et al., 1991 | PROMISE | ACM | Milrinone | Placebo | 1088 | 235 (21,6) | 21 | 0 | 100 | 54 |
| Packer et al., 1993 | RADIANCE study | Worsening HF | Digoxin | Placebo | 178 | 42 (23,6) | 27 | 73 | 27 | - |
| Packer et al., 1996 | PRAISE | ACM | Amlodipine | Placebo | 1153 | 278 (24,1) | 21 | 0 | 100 | 63 |

**Supplementary table S2** *Continued*

| **First authors, year** | **Study abbreviation** | **Primary endpoint** | **Treatment** | **Control treatment** | **N patients** | **Women (%)** | **LVEF** | **NYHA class I/II** | **NYHA class III/IV** | **Ischemic etiology** |
| --- | --- | --- | --- | --- | --- | --- | --- | --- | --- | --- |
| Packer et al., 1996 | U.S. Carvedilol Heart Failure Study Group | ACM | Carvedilol | Placebo | 1094 | 256 (23,4) | 23 | 53 | 47 | - |
| Packer et al., 1999 | ATLAS | ACM | Lisinopril (2.5-5.0 mg) | Lisinopril (32.5 -35 mg) | 3194 | 678 (21,2) | 23 | 16 | 84 | - |
| Packer et al., 2001 | COPERNICUS | ACM | Carvedilol | Placebo | 2289 | 470 (20,5) | 20 | - | - | 67 |
| Packer et al., 2002 | OVERTURE | Combined (ACM, HFH requiring IV treatment) | Enalapril | Omapatrilat | 5770 | 1211 (21) | 24 | 48 | 52 | 56 |
| Packer et al., 2017 | PROFILE | ACM | Flosequinan | Placebo | 2345 | 494 (21,1) | 23 | 0 | 100 | 54 |
| Packer et al., 2017 | TRUE-AHF | CV mortality | Ularitide IV | Placebo | 2157 | 737 (34,2) | - | 38 | 62 | - |
| Packer et al., 2017 | ENABLE  (trial 1 and 2) | Combined (ACM, HFH) | Bosentan | Placebo | 1611 | 414 (25,7) | 25 | 0 | 100 | 69 |
| Packer et al., 2020 | EMPEROR-Reduced | Combined (CV mortality, HFH) | Empagliflozin | Placebo | 3730 | 893 (23,9) | 27 | 75 | 25 | 52 |
| Packer et al., 2024 | SUMMIT | Combined (CV death, worsening HF) | Tirzepatide | Placebo | 731 | 338 | 61 | 73 | 27 | - |
| Paterna et al., 2011 | SMAC-HF study | ACM | Furosemide with HSS | Furosemide without HSS | 1927 | 714 (37,1) | 35 | - | - | - |
| Pfeffer et al., 2003 | Valsartan in Acute Myocardial Infarction Trial | ACM | I. Valsartan  II. Valsartan + Captopril | I. and II. Captopril | 14703 | 4570 (31,1) | 35 | 76 | 24 | 100 |
| Piccini et al., 2019 | GENETIC-AF | Combined (ACM, AFL) | Bucindolol | Metoprolol | 267 | 48 (18) | 36 | 85 | 15 | 32 |
| Pitt et al., 1999 | RALES | ACM | Spironolactone | Placebo | 1663 | 446 (26,8) | 25 | 0 | 100 | 55 |
| Pitt et al., 2000 | Losartan Heart Failure Survival Study ELITE II | ACM | Losartan | Captopril | 3152 | 967 (30,7) | 31 | 52 | 48 | 79 |
| Pitt et al., 2003 | EPHESUS group | Combined (ACM, CV mortality, HFH, MI, stroke, ventricular arrhythmia) | Eplerenone | Placebo | 6632 | 1918 (28,9) | 33 | 52 | 48 | 100 |
| Pitt et al., 2014 | TOPCAT | Combined (CV mortality, aborted cardiac arrest, HFH) | Spironolactone | Placebo | 3445 | 1775 (51,5) | 56* | 67 | 33 | - |
| Ponikowski et al., 2020 | AFFIRM-AHF | Combined (CV mortality and total HFH) | Ferric carboxymaltose IV | Placebo | 1108 | 494 (44,6) | 33 | 47 | 53 | 47 |
| Poole-Wilson et al., 2003 | COMET | ACM | Carvedilol | Metoprolol | 3029 | 612 (20,2) | 26 | 48 | 52 | 53 |
| Sakata et al., 2015 | SUPPORT | Combined (ACM, non-fatal MI or stroke and HFH) | Olmesartan | Placebo | 1147 | 291 (25,4) | 54 | 93 | 7 | 48 |

**Supplementary table S2** *Continued*

| **First authors, year** | **Study abbreviation** | **Primary endpoint** | **Treatment** | **Control Treatment** | **N patients** | **Women (%)** | **LVEF** | **NYHA class I/II** | **NYHA class III/IV** | **Ischemic etiology** |
| --- | --- | --- | --- | --- | --- | --- | --- | --- | --- | --- |
| Shchekochikhin et al., 2023 |  | Time to AKI | Dapagliflozin | Usual Care | 200 | 98 (49) | 47 | - | - | 47 |
| Singh et al., 1995 | Survival Trial of Antiarrhytmitc Therapy in Congestive Heart failure | ACM | Amiodarone | Placebo | 674 | 7 (1) | - | 54 | 41 | 71 |
| Sliwa et al., 2016 | BA-HEF | Combined (ACM, HFH) | Hydralazine-isosorbide dinitrate | Placebo | 133 | 65 (49,2) | 25 | 23 | 77 | 5 |
| Solomon et al., 2019 | PARAGON-HF | Combined (total HFH, CV mortality) | Sacubitril-valsartan | Valsartan | 4796 | 2479 (51,7) | 58 | 80 | 20 | 36 |
| Solomon et al., 2024 | FINEARTS-HF | Combined (CV death, Worsening HF) | Finerenone | Placebo | 6001 | 2732 (45,5) | 53 | 69 | 31 | - |
| Solomon et al., 2022 | DELIVER | Combined (worsening HF, CV mortality) | Dapagliflozin | Placebo | 6263 | 2747 (43,9) | 54 | 75 | 25 |  |
| Sturm et al., 2000 |  | Combined (ACM, worsening HF) | Atenolol | Placebo | 100 | 12 (12) | 17 | 78 | 22 | 28 |
| Suzuki et al., 2021 |  | CV events | Allopurinol | Febuxostat | 263 | 88 (33,5) | 55 | 95 | 4 | 32 |
| Swedberg et al., 2010 | SHIFT | Combined (CV mortality, HFH) | Ivabradine | Placebo | 6505 | 1535 (23,6) | 29 | 49 | 51 | 68 |
| Swedberg et al., 2013 | RED-HF | Combined (ACM, HFH) | Darbepoetin alfa | Placebo | 2278 | 944 (41,4) | 31* | 35 | 65 | 73 |
| Takano et al., 2013 | PEARL study | Combined (CV mortality, HFH) | Pitavastatin | No statin | 574 | 106 (18,5) | 34 | 90 | 10 | 27 |
| Tavazzi et al., 2008 | GISSI-HF | ACM | n-3 PUFA | Placebo | 6975 | 1516 (21,7) | 33 | 63 | 61 | 50 |
| Tavazzi et al., 2008 | GISSI-HF | ACM | Rosuvastatin | Placebo | 4574 | 1032 (22,6) | 33 | 63 | 37 | 40 |
| Teerlink et al., 2021 | GALACTIC-HF | Combined (CV mortality, first HF event) | Omecamtiv mecarbil | Placebo | 8232 | 1749 (21,2) | 27 | 53 | 47 | 54 |
| The Digitalis Investigation Group, 1997 | DIG | ACM | Digoxin | Placebo | 6800 | 1519 (22,3) | 29 | 67 | 33 | 71 |
| Torp-Pedersen et al., 1999 |  | ACM | Dofetilide | Placebo | 1518 | 404 (26,6) | - | 39 | 60 | - |
| Torp-Pedersen et al., 2008 |  | Combined (ACM, HFH) | Nolomirole | Placebo | 1000 | 265 (26,5) | - | 45 | 55 | - |
| Torre-Amione et al., 2008 | ACCLAIM | Combined (ACM, first hospitalization for CV reasons) | Immunomodulation therapy | Placebo | 2408 | 476 (19,8) | 23 | 29 | 71 | 68 |

**Supplementary table S2** *Continued*

| **First authors, year** | **Study abbreviation** | **Primary endpoint** | **Treatment** | **Control Treatment** | **N patients** | **Women (%)** | **LVEF** | **NYHA class I/II** | **NYHA class III/IV** | **Ischemic etiology** |
| --- | --- | --- | --- | --- | --- | --- | --- | --- | --- | --- |
| Triposkiadis et al., 2014 | DAD-HF II | ACM | I. Furosemide (high-dose)  II. Furosemide (low-dose) + dopamine | I. Furosemide (low-dose) + dopamine  II. Furosemide (low-dose) | 161 | 74 (46) | 32 | - | - | - |
| Tsutsui et al., 2017 | J-EMPHASIS-HF | Combined (CV mortality, HFH) | Eplerenone | Placebo | 221 | 45 (20,4) | 26 | 83 | 17 | 74 |
| Tsutsui et al., 2019 | J-SHIFT | Combined (CV mortality, HFH) | Ivabradine | Placebo | 254 | 45 (17,7) | 27 | 80 | 21 | 39 |
| Vrtovec et al., 2008 |  | SCD | Atorvastatin | Placebo | 110 | 43 (39,1) | 25 | - | - | 59 |
| Waagenstein et al., 1993 | MDC | Combined (ACM or need for heart transplantation) | Metoprolol | Placebo | 383 | 105 (27,4) | 22 | 47 | 52 | - |
| Wang et al., 2017 |  | ACM | Traditional Chinese Medicine | Placebo | 334 | 151 (45,2) | - | 65 | 89 | 80 |
| Willenheimer et al., 2005 | CIBIS-III | Combined (ACM or any hospitalization) | Bisoprolol | Enalapril | 1010 | 321 (31,8) | 29 | 49 | 51 | 62 |
| Wojnicz et al., 2006 |  | Combined (ACM, urgent heart transplantation, HFH) | Enoxaparin | Usual Care | 102 | 15 (14,7) | 29 | 88 | 12 | 0 |
| Wu et al., 2016 |  | ACM | Spironolactone | Usual Care | 139 | 69 (49,6) | 32 | 100 | 0 | - |
| Yamamoto et al., 2013 | J-DHF | Combined (CV mortality, HFH) | Carvedilol | Placebo | 245 | 103 (42) | 63 | 91 | 9 | 15 |
| Yancy et al., 2008 | FUSION II | Combined (ACM, CV or renal hospitalization) | Nesiritide IV | Placebo | 911 | 264 (29) | 25 | 0 | 100 | 64 |
| Yin et al., 2022 |  | Delirium | Melatonin | Placebo | 497 | 202 (40,6) | - | - | - | - |
| Young et al., 2004 | CHARM low-left | Combined (CV mortality, HFH) | Candesartan | Placebo | 4576 | 1188 (26) | 29 | 35 | 65 | - |
| Yusuf et al., 1991 | SOLVD-treatment | ACM | Enalapril | Placebo | 2569 | 504 (19,6) | 25 | 68 | 32 | 71 |
| Yusuf et al., 1992 | SOLVD-prevention | ACM | Enalapril | Placebo | 4228 | 484 (11,4) | 28 | 100 | 0 | 83 |
| Yusuf et al., 2003 | CHARM-Preserved Trial | Combined (CV mortality, HFH) | Candesartan | Placebo | 3023 | 1212 (40,1) | 54 | 61 | 39 | 56 |
| Zannad et al., 1998 |  | Combined (ACM, HFH) | Fosinopril | Enalapril | 254 | 56 (22) | 29 | 82 | 18 | 69 |
| Zannad et al., 2011 | EMPHASIS-HF | Combined (CV mortality, HFH) | Eplerenone | Placebo | 2737 | 610 (22,3) | 26 | 100 | 0 | 69 |
| Zannad et al., 2018 | COMMANDER HF | Combined (ACM, MI, stroke) | Rivaroxaban | Placebo | 5022 | 1150 (22,9) | 34 | 47 | 53 | - |
| Zitterman et al., 2017 | EVITA | ACM | Vitamin D | Placebo | 400 | 68 (17) | 28 | 73 | 27 | 45 |

*Median; Abbrevations used: ACM = all-cause mortality; HFH = heart failure hospitalisation; HF = heart failure, CV = cardiovascular; MI = myocardial infarction; AP = angina pectoris; CHF = congestive heart failure; IV = intravenous; AFL = atrium fibrillation / flutter; AKI = acute kidney injury; SCD = sudden cardiac death; HFF = Hypertonic saline solution

## Supplementary table S3 Sex specific in and exclusion criteria

| **First author** | **Year** | **Sex-specific in and exclusion criteria** |
| --- | --- | --- |
| Angermann et al. | 2016 | Pregnancy or lactation |
| Anker et al. | 2021 | Pregnancy or lactation |
| Armstrong et al. | 2020 | Women: unwillingness to use medically accepted contraception |
| Bhatt et al. | 2021 | Pregnancy, lactation, or unwillingness to use medically accepted contraception; upper age limit (max. 85 years old) |
| Borghi et al. | 2012 | Pregnancy |
| Borghi et al. | 2013 | Upper age limit (max. 90 years old) |
| Brown et al. | 1995 | Pregnancy, lactation, or unwillingness to use medically accepted contraception. |
| Cheang et al. | 2024 | Pregnancy, lactation, or unwillingness to use medically accepted contraception. |
| CIBIS-II investigators and committees | 1999 | Women only if premenopausal; exclusion if not surgically sterilized or unwillingness to use medically accepted contraception |
| Clement et al. | 2000 | Pregnancy or lactation |
| Cohn et al. | 1998 | Postpartum <60 days, unwillingness to use medically accepted contraception. |
| Cohn et al. | 2001 | Pregnancy, lactation, or unwillingness to use medically accepted contraception; postpartum cardiomyopathy. |
| Cokkinos et al. | 2006 | Upper age limit (max. 75 years old) |
| Cosín-Aguilar et al. | 1999 | Women with childbearing potential |
| Dargie et al. | 2001 | Pregnancy or lactation |
| EPOCH Study Group | 2002 | Pregnancy, lactation or women of childbearing potential who are not sterilized |
| Fox et al. | 2008 | Pregnancy, lactation or childbearing potential. |
| Gheorghiade et al. | 2004 | Pregnancy or lactation. |
| Hampton et al. | 1997 | Pregnancy, lactation, or unwillingness to use medically accepted contraception. |
| Hoeper et al. | 2024 | Pregnancy and breastfeeding; both fertile men and women: medically accepted contraception |
| Holubarch et al. | 2008 | Pregnancy, lactation, or unwillingness to use medically accepted contraception. |
| Homma et al. | 2012 | Pregnancy or unwillingness to use medically accepted contraception. |
| Honda et al. | 2024 | Possibility for pregnancy |
| Jia et al. | 2014 | Childbearing potential |
| Karla et al. | 2022 | Pregnancy or unwillingness to use medically accepted contraception. |
| Kjøller-Hansen et al. | 2000 | Childbearing potential; upper age limit (max. 75 years old) |
| Kleber et al. | 1992 | Pregnancy or lactation |

**Supplementary table S3** *Continued*

| **First author** | **Year** | **Sex-specific in and exclusion criteria** |
| --- | --- | --- |
| Køber et al. | 1995 | Pregnancy or lactation |
| Køber et al. | 2000 | Childbearing potential |
| Køber et al. | 2008 | Pregnancy or lactation |
| Konstam et al. | 2007 | Pregnancy or unwillingness to use medically accepted contraception. |
| Konstam et al. | 2009 | Pregnancy or lactation |
| Kozhuharov et al. | 2019 | Pregnancy |
| Lechat et al. | 1994 | Upper age limit (max. 75 years old) |
| Massie et al. | 2009 | Pregnancy, lactation or had childbearing potential. |
| Masuyama et al. | 2012 | Pregnancy |
| Matsumori et al. | 2003 | Pregnancy, lactation or had childbearing potential. |
| MERIT-HF study group | 1999 | Upper age limit (max. 80 years old) |
| McMurray et al. | 2019 | Pregnancy, lactation, or unwillingness to use medically accepted contraception. |
| McMurray et al. | 2007 | Pregnancy, lactation, or unwillingness to use medically accepted contraception. |
| McMurray et al. | 2016 | Pregnancy, lactation, or unwillingness to use medically accepted contraception. |
| Mentz et al. | 2023 | (Planning to get) pregnant or lactation |
| Metra et al. | 2019 | Pregnancy, lactation, or unwillingness to use medically accepted contraception. |
| Miller et al. | 2008 | Pregnancy |
| Moiseyev et al. | 2002 | Women with childbearing potential |
| Mortensen et al. | 2014 | Women with childbearing potential and lactation |
| Mullens et al. | 2022 | Pregnancy or lactation |
| NETWORK investigators | 1998 | Upper age limit (max. 85 years old) |
| O'Connor et al. | 2011 | Pregnancy or lactation |
| O'Connor et al. | 2003 | Pregnancy or unwillingness to use medically accepted contraception. |
| Packer et al. | 2017 | Pregnancy or lactation; upper age limit (max. 85 years old). |
| Packer et al. | 2020 | Pregnancy, lactation, or unwillingness to use medically accepted contraception. |
| Packer et al. | 2025 | Fertile males: with partners of childbearing potentials who are unwilling to use medically accepted contraception; Women: pregnancy, lactation or unwillingness to use medically acepted contraception |
| Pfeffer et al. | 2003 | Pregnancy, lactation, or unwillingness to use medically accepted contraception. |

**Supplementary table S3** *Continued*

| **First author** | **Year** | **Sex-specific in and exclusion criteria** |
| --- | --- | --- |
| Piccini et al. | 2019 | Upper age limit (max. 85 years old) |
| Pitt et al. | 2014 | Pregnancy, lactation, or unwillingness to use medically accepted contraception. |
| Ponikowski et al. | 2020 | Pregnancy, lactation, or unwillingness to use medically accepted contraception |
| Poole-Wilson et al. | 2003 | Pregnancy or unwillingness to use medically accepted contraception. |
| Sakata et al. | 2015 | Pregnancy; upper age limit (max. 80 years old) |
| Shchekochikhin et al. | 2023 | Pregnancy or lactation |
| Singh et al. | 1995 | Women of childbearing potential |
| Sliwa et al. | 2016 | Pregnancy or lactation |
| Solomon et al. | 2022 | Pregnancy, lactation, or unwillingness to use medically accepted contraception. |
| Solomon et al. | 2019 | Pregnancy, lactation, or unwillingness to use medically accepted contraception. |
| Solomon et al. | 2024 | Pregnancy, lactation, or unwillingness to use medically accepted contraception. History of peripartum cardiomyopathy |
| Sturm et al. | 2000 | Pregnancy or unwillingness to use medically accepted contraception; upper age limit (max. 75 years old) |
| Swedberg et al. | 2013 | Pregnancy, lactation, or unwillingness to use medically accepted contraception. |
| Takano et al. | 2013 | Pregnancy; upper age limit (max. 79 years old) |
| Tavazzi et al. | 2008 | Pregnancy, lactation, or unwillingness to use medically accepted contraception. |
| Tavazzi et al. | 2008 | Pregnancy, lactation, or unwillingness to use medically accepted contraception. |
| Teerlink et al. | 2021 | Pregnancy, lactation, or unwillingness to use **two** medically accepted contraception/ male should inform their female partner; upper age limit (max. 85 years old) |
| Torp-Pedersen et al. | 1999 | Childbearing potential |
| Torp-Pedersen et al. | 2008 | Pregnancy or unwillingness to use medically accepted contraception |
| Torre-Amione et al. | 2008 | Pregnancy or unwillingness to use medically accepted contraception |
| Waagstein et al. | 1993 | Upper age limit (max. 75 years old) |
| Wang et al. | 2017 | Pregnancy or lactation; upper age limit (max. 79 years old) |
| Wu et al. | 2016 | Pregnancy or lactation; upper age limit (max. 80 years old) |
| Yusuf et al. | 1991 | Pregnancy or unwillingness to use medically accepted contraception; upper age limit (max. 80 years old) |
| Yusuf et al. | 1992 | Pregnancy or unwillingness to use medically accepted contraception; upper age limit (max. 80 years old) |
| Zannad et al. | 2018 | Women: Pregnancy, lactation, or unwillingness to use medically accepted contraception; wanting to donate eggs for assisted reproduction; Men: barrier methods (if sexually active with females of childbearing potential); wanting to donate sperm |
| Zitterman et al. | 2017 | Upper age limit (max. 80 years old) |

## Supplementary table S4 Sex-specific relative effect measures of the primary endpoint

| **Trial** | **Measure** | **REM men (95% CI)** | **REM women (95% CI)** | **Ratio**  **(REM_women_/REM_men_)** | **Delta ln(REM)**  **(95% CI)** |
| --- | --- | --- | --- | --- | --- |
| **Beta-blocker** |  |  |  |  |  |
| U.S. Carvedilol Heart Failure Study Group (1996) | HR | 0,41 (0,22 - 0,80) | 0,23 (0,07 - 0,69) | 0,56 | 0,58 (-0,74 - 1,89) |
| CIBIS-II (1999) | OR | 0,67 (0,53 - 0,85) | 0,48 (0,26 - 0,87) | 0,72 | 0,33 (-0,31 - 0,98) |
| MERIT-HF (1999) | RR | 0,61 (0,49 - 0,78) | 0,92 (0,56 - 1,50) | 1,51 | -0,41 (-0,96 - 0,13) |
| BEST (2001) | HR | 0,90 (0,77 - 1,05) | 0,90 (0,65 - 1,22) | 1,00 | 0,00 (-0,35 - 0,35) |
| COPERNICUS (2001) | HR | 0,65 (0,51 - 0,84) | 0,65 (0,39 - 1,11) | 1,00 | 0,00 (-0,58 - 0,58) |
| COMET (2003) | HR | 0,80 (0,7 - 0,91) | 0,97 (0,73 - 1,27) | 1,21 | -0,19 (-0,50 - 0,11) |
| CIBIS-III (2005) | HR | 0,94 (0,74 - 1,20) | 0,96 (0,65 - 1,39) | 1,02 | -0,02 (-0,47 - 0,43) |
| SENIORS (2005) | HR | 0,93 (0,78 - 1,11) | 0,72 (0,55 - 0,94) | 0,77 | 0,26 (-0,06 - 0,58) |
| J-DHF (2013) | HR | 0,82 (0,43 - 1,59) | 1,02 (0,47 - 2,21) | 1,24 | -0,21 (-1,23 - 0,80) |
| **RAAS-inhibition** |  |  |  |  |  |
| CONSENSUS (1987) | RR | 0,61 (0,44 – 0,85) | 1,14 (0,68 – 0,90) | 1,87 | -0,63 (-0,98 - -0,27) |
| SOLVD-treatment (1991) | RR | 0,89 (0,80 – 0,99) | 0,86 (0,67 – 1,09) | 0,97 | 0,03 (-0,23 - 0,30) |
| SOLVD-prevention (1992) | RR | 0,90 (0,77 – 1,05) | 1,15 (0,74 – 1,78) | 1,28 | -0,25 (-0,71 - 0,22) |
| AIRE (1993) | HR | 0,75 (0,59 - 0,97) | 0,68 (0,48 - 0,96) | 0,91 | 0,10 (-0,33 - 0,52) |
| TRACE (1995) | RR | 0,74 (0,62 - 0,89) | 0,90 (0,69 - 1,18) | 1,22 | -0,20 (-0,52 - 0,13) |
| ATLAS (1999) | HR | 0,89 (0,79 - 1,00) | 1,06 (0,83 - 1,35) | 1,19 | -0,17 (-0,44 - 0,10) |
| Losartan Heart Failure Survival Study ELITE II (2000) | HR | 1,12 (0,92 - 1,36) | 1,14 (0,83 - 1,59) | 1,02 | -0,02 (-0,40 - 0,36) |
| Val-HeFT (2001) | RR | 0,87 (0,77 - 0,98) | 0,83 (0,66 - 1,05) | 0,95 | 0,05 (-0,21 - 0,31) |
| OVERTURE (2002) | HR | 0,96 (0,88 - 1,05) | 0,90 (0,77 - 1,07) | 0,94 | 0,06 (-0,13 - 0,25) |
| Valsartan in Acute Myocardial Infarction Trial (2003) | HR | 1,03 (0,91 - 1,16) | 0,96 (0,82 - 1,12) | 0,93 | 0,07 (-0,13 - 0,27) |
| Valsartan in Acute Myocardial Infarction Trial (2003) | HR | 1,01 (0,89 - 1,14) | 0,94 (0,81 - 1,10) | 0,93 | 0,07 (-0,12 - 0,27) |
| CHARM low-left (2004) | HR | 0,82 (0,74 - 0,91) | 0,82 (0,68 - 0,99) | 0,99 | 0,01 (-0,21 - 0,22) |
| PEP-CHF (2006) | HR | 0,73 (0,41 - 1,31) | 0,67 (0,40 - 1,10) | 0,92 | 0,09 (-0,68 - 0,86) |
| I-PRESERVE (2008) | HR | 0,96 (0,83 - 1,12) | 0,94 (0,82 - 1,08) | 0,98 | 0,02 (-0,18 - 0,22) |
| HEAAL study (2009) | HR | 0,86 (0,77 - 0,96) | 1,02 (0,85 - 1,23) | 1,19 | -0,17 (-0,39 - 0,04) |
| ASTRONAUT (2013) | HR | 0,89 (0,72 - 1,10) | 1,02 (0,66 - 1,57) | 1,15 | -0,14 (-0,62 - 0,35) |
| Borghi et al. (2013) | OR | 0,57 (0,30 - 0,98) | 0,66 (0,30 - 1,46) | 1,16 | -0,15 (-1,13 - 0,84) |
| PARADIGM-HF (2014) | HR | 0,81 (0,73 - 0,89) | 0,77 (0,62 - 0,94) | 0,94 | 0,06 (-0,17 - 0,29) |
| SUPPORT (2015) | HR | 1,22 (0,96 - 1,55) | 1,07 (0,69 - 1,66) | 0,88 | 0,13 (-0,36 - 0,63) |

**Supplementary table S4.** *Continued*

| **Trial** | **Measure** | **REM male (95% CI)** | **REM female (95% CI)** | **Ratio**  **(REM_female_/REM_male_)** | **Delta ln(REM)**  **(95% CI)** |
| --- | --- | --- | --- | --- | --- |
| ATMOSPHERE (2016) | HR | 0,92 (0,82 - 1,02) | 0,99 (0,78 - 1,25) | 1,07 | -0,07 (-0,33 - 0,19) |
| ATMOSPHERE (2016) | HR | 0,95 (0,85 - 1,06) | 1,21 (0,96 - 1,52) | 1,27 | -0,24 (-0,49 - 0,01) |
| PARAGON-HF (2019) | HR | 1,03 (0,85 - 1,25) | 0,73 (0,59 - 0,90) | 0,71 | 0,34 (0,06 - 0,63) |
| **MRA** |  |  |  |  |  |
| RALES (1999) | RR | 0,70 (0,59 - 0,84) | 0,71 (0,52 - 0,97) | 1,01 | -0,01 (-0,37 - 0,34) |
| [EPHESUS group (2003)](https://www.nejm.org/doi/full/10.1056/NEJMoa030207#FN1) | RR | 0,86 (0,75 - 1,04) | 0,79 (0,66 - 0,97) | 0,92 | 0,08 (-0,17 - 0,34) |
| EMPHASIS-HF (2011) | HR | 0,73 (0,62 - 0,86) | 0,61 (0,43 - 0,86) | 0,84 | 0,18 (-0,20 - 0,56) |
| TOPCAT (2014) | HR | 0,89 (0,73 - 1,09) | 0,89 (0,71 - 1,12) | 1,00 | 0,00 (-0,30 - 0,30) |
| J-EMPHASIS-HF (2017) | HR | 0,96 (0,57 - 1,61) | 0,72 (0,23 - 2,23) | 0,75 | 0,29 (-0,96 - 1,54) |
| EARLIER trial (2022) | HR | 1,25 (0,70 - 2,23) | 1,07 (0,30 - 3,82) | 0,86 | 0,15 (-1,24 - 1,54) |
| FINEARTS-HF (2024) | RR | 0,88 (0,74 - 1,04) | 0,78 (0,65 - 0,95) | 0,89 | 0,12 (-0,13 - 0,38) |
| **SGLT-2 inhibition** |  |  |  |  |  |
| DAPA-HF (2019) | HR | 0,73 (0,63 - 0,85) | 0,79 (0,59 - 1,06) | 1,08 | -0,08 (-0,41 - 0,25) |
| EMPEROR-Reduced (2020) | HR | 0,80 (0,68 - 0,93) | 0,59 (0,44 - 0,80) | 0,74 | 0,30 (-0,03 - 0,64) |
| EMPEROR-Preserved (2021) | HR | 0,81 (0,69 - 0,96) | 0,75 (0,61 - 0,92) | 0,93 | 0,08 (-0,19 - 0,34) |
| SOLOIST-WHF (2021) | HR | 0,62 (0,47 - 0,82) | 0,80 (0,51 - 1,25) | 1,29 | -0,25 (-0,78 - 0,27) |
| DELIVER (2022) | HR | 0,82 (0,71 - 0,96) | 0,81 (0,67 - 0,97) | 0,99 | 0,01 (-0,23 - 0,25) |
| **Inotropics and/or vasodilators** |  |  |  |  |  |
| PROMISE (1991) | HR | 1,26 (0,97 - 1,63) | 1,33 (0,79 - 2,23) | 1,06 | -0,05 (-0,63 - 0,53) |
| Feldman et al. (1993) | OR | 0,44 (0,25 - 0,75) | 0,70 (0,14 - 3,41) | 1,59 | -0,46 (-2,15 - 1,22) |
| PRIME II (1997) | OR | 1,31 (1,00 - 1,71) | 0,86 (0,49 - 1,51) | 0,66 | 0,42 (-0,20 - 1,04) |
| Vesnarinone Trial Investigators (2003) | HR | 1,20 (1,00 - 1,43) | 1,32 (0,92 - 1,94) | 1,11 | -0,10 (-0,51 - 0,31) |
| SURVIVE (2007) | HR | 0,93 (0,73 - 1,19) | 0,89 (0,60 - 1,62) | 0,96 | 0,04 (-0,51 - 0,60) |
| ESSENTIAL (2009) | HR | 0,98 (0,85 - 1,25) | 0,99 (0,74 - 1,32) | 1,01 | -0,01 (-0,36 - 0,34) |
| Warcef (2012) | HR | 0,88 (0,73 - 1,05) | 1,34 (0,88 - 2,03) | 1,52 | -0,42 (-0,88 - 0,04) |
| GALACTIC (2019) | HR | 0,85 (0,61 - 1,19) | 1,67 (1,08 - 2,59) | 1,96 | -0,68 (-1,23 - -0,12) |
| VICTORIA (2020) | HR | 0,90 (0,81 - 1,00) | 0,88 (0,73 - 1,08) | 0,98 | 0,02 (-0,20 - 0,24) |
| GALACTIC-HF (2021) | HR | 0,92 (0,85 - 0,99) | 0,95 (0,81 - 1,12) | 1,03 | -0,03 (-0,21 - 0,15) |
| LASCAR-AHF trial (2024) | HR | 0,99 (0,55 - 1,81) | 1,38 (0,66 - 2,89) | 1,39 | -0,33 (-1,28 - 0,62) |

**Supplementary table S4.** *Continued*

| **Trial** | **Measure** | **REM male (95% CI)** | **REM female (95% CI)** | **Ratio**  **(REM_female_/REM_male_)** | **Delta ln(REM)**  **(95% CI)** |
| --- | --- | --- | --- | --- | --- |
| **Diuretics** |  |  |  |  |  |
| EVEREST outcome trial (2007) | HR | 1,03 (0,89 - 1,18) | 0,87 (0,67 - 1,12) | 0,84 | 0,17 (-0,12 - 0,46) |
| J-MELODIC (2012) | HR | 0,44 (0,23 - 0,84) | 0,90 (0,35 - 2,29) | 2,05 | -0,72 (-1,86 - 0,43) |
| ADVOR (2022) | RR | 1,67 (1,24 - 2,25) | 1,21 (0,86 - 1,71) | 0,72 | 0,32 (-0,13 - 0,78) |
| TRUE-AHF (2017) | HR | 1,01 (0,81 - 1,26) | 1,08 (0,78 - 1,50) | 1,07 | -0,07 (-0,46 - 0,33) |
| TRANSFORM-HF (2023) | HR | 1,06 (0,88 - 1,27) | 0,97 (0,77 - 1,22) | 0,92 | 0,09 (-0,21 - 0,38) |
| **Others** |  |  |  |  |  |
| GESICA (1994) | OR | 0,78 (0,53 - 1,16) | 0,48 (0,20 - 1,13) | 0,62 | 0,49 (-0,46 - 1,44) |
| PRAISE (1996) | HR | 0,92 (0,74 - 1,15) | 0,62 (0,40 - 0,96) | 0,67 | 0,39 (-0,10 - 0,88) |
| DIG (1997) | HR | 0,95 (0,87 - 1,04) | 1,17 (0,97 - 1,40) | 1,23 | -0,21 (-0,41 - 0,00) |
| Torp-Pedersen et al. (1999) | HR | 1,00 (0,83 - 1,20) | 0,85 (0,62 - 1,15) | 0,85 | 0,16 (-0,20 - 0,52) |
| DIAMOND-MI (2000) | HR | 0,94 (0,76 - 1,17) | 0,92 (0,66 - 1,28) | 0,98 | 0,02 (-0,37 - 0,42) |
| SCD-HeFT (2005) | HR | 1,04 (0,83 - 1,30) | 1,17 (0,72 - 1,90) | 1,13 | -0,12 (-0,65 - 0,42) |
| CORONA (2007) | HR | 0,94 (0,84 - 1,06) | 0,86 (0,68 - 1,09) | 0,91 | 0,09 (-0,17 - 0,35) |
| ACCLAIM (2008) | HR | 0,96 (0,82 - 1,11) | 0,78 (0,56 - 1,09) | 0,81 | 0,21 (-0,16 - 0,57) |
| BEAUTIFUL (2008) | HR | 0,98 (0,88 - 1,08) | 1,14 (0,90 - 1,44) | 1,16 | -0,15 (-0,41 - 0,11) |
| SHIFT (2010) | HR | 0,84 (0,76 - 0,94) | 0,74 (0,60 - 0,91) | 0,88 | 0,13 (-0,11 - 0,36) |
| RED-HF (2013) | HR | 1,01 (0,87 - 1,16) | 0,96 (0,79 - 1,18) | 0,95 | 0,05 (-0,20 - 0,30) |
| Q-SYMBIO (2014) | HR | 0,54 (0,33 - 0,89) | 0,49 (0,15 - 1,62) | 0,91 | 0,10 (-1,19 - 1,39) |
| MOOD-HF (2016) | HR | 1,01 (0,75 - 1,36) | 0,91 (0,54 - 1,57) | 0,90 | 0,10 (-0,51 - 0,72) |
| ENABLE trial 1 and 2 (2017) | HR | 0,93 (0,74 - 1,14) | 0,82 (0,68 - 0,99) | 0,88 | 0,13 (-0,16 - 0,41) |
| COMMANDER HF (2018) | HR | 0,92 (0,81 - 1,04) | 1,05 (0,83 - 1,33) | 1,14 | -0,13 (-0,40 - 0,13) |
| J-SHIFT (2019) | HR | 0,73 (0,42 - 1,72) | 0,46 (0,12 - 1,86) | 0,63 | 0,46 (-1,08 - 2,00) |
| RELAX-AHF-2 (2019) | HR | 0,91 (0,74 - 1,13) | 1,08 (0,83 - 1,40) | 1,19 | -0,17 (-0,51 - 0,17) |
| AFFIRM-AHF (2020) | RR | 0,64 (0,46 - 0,89) | 1,05 (0,72 - 1,53) | 1,64 | -0,50 (-1,00 - 0,01) |
| IRONMAN (2022) | RR | 0,81 (0,63 - 1,03) | 0,86 (0,53 - 1,38) | 1,06 | -0,06 (-0,60 - 0,48) |
| QUEST (2024) | HR | 0,76 (0,65 - 0,90) | 0,79 (0,59 - 1,05) | 1,04 | -0,04 (-0,37 - 0,29) |
| SUMMIT (2025) | HR | 0,61 (0,33 - 1,13) | 0,66 (0,38 - 1,18) | 1,08 | -0,08 (-0,92 - 0,76) |

## Supplementary figure S1 Risk of bias analysis per study.

## Supplementary figure S2 Risk of bias analysis - summary figure.

## Supplementary figure S3 Funnel plot.

Supplementary figure S4A+B Bubble plots: absolute number of women

## Supplementary figure S5A-C Bubble plots: absolute number of women

## Supplementary figure S6 Bubble plot: left ventricular ejection fraction

## Supplementary figure S7 Bubble plot: serious adverse events

## Supplementary references

1.	Abraham WT, Anand IS, Klapholz M, Ponikowski P, Scarlata D, Wasserman SM, et al. Treatment of anemia with darbepoetin alfa in heart failure. Congest Heart Fail. 2010;16(3):87-95.

2.	Altenberger J, Parissis JT, Costard-Jaeckle A, Winter A, Ebner C, Karavidas A, et al. Efficacy and safety of the pulsed infusions of levosimendan in outpatients with advanced heart failure (LevoRep) study: a multicentre randomized trial. Eur J Heart Fail. 2014;16(8):898-906.

3.	Anand I, McMurray J, Cohn JN, Konstam MA, Notter T, Quitzau K, et al. Long-term effects of darusentan on left-ventricular remodelling and clinical outcomes in the EndothelinA Receptor Antagonist Trial in Heart Failure (EARTH): randomised, double-blind, placebo-controlled trial. Lancet. 2004;364(9431):347-54.

4.	Anderson JL, Krause-Steinrauf H, Goldman S, Clemson BS, Domanski MJ, Hager WD, et al. Failure of benefit and early hazard of bucindolol for Class IV heart failure. J Card Fail. 2003;9(4):266-77.

5.	Anonymous. Xamoterol in severe heart failure. The Xamoterol in Severe Heart Failure Study Group. Lancet. 1990;336(8706):1-6.

6.	Anonymous. Randomised, placebo-controlled trial of carvedilol in patients with congestive heart failure due to ischaemic heart disease. Australia/New Zealand Heart Failure Research Collaborative Group. Lancet. 1997;349(9049):375-80.

7.	Bartunek J, Terzic A, Davison BA, Filippatos GS, Radovanovic S, Beleslin B, et al. Cardiopoietic cell therapy for advanced ischaemic heart failure: Results at 39 weeks of the prospective, randomized, double blind, sham-controlled CHART-1 clinical trial. Eur Heart J. 2017;38(9):648-60.

8.	Bergler-Klein J, Sochor H, Pouleur H, Pacher R, Porenta G, Glogar D. Safety of concomitant potassium-sparing diuretics in angiotensin-converting enzyme inhibitor therapy in severe congestive heart failure. Xamoterol in Severe Heart Failure Study Group. J Cardiovasc Pharmacol. 1994;24(2):194-8.

9.	Boccanelli A, Mureddu GF, Cacciatore G, Clemenza F, Di Lenarda A, Gavazzi A, et al. Anti-remodelling effect of canrenone in patients with mild chronic heart failure (AREA IN-CHF study): Final results. Eur J Heart Fail. 2009;11(1):68-76.

10.	Butler J, Anker SD, Lund LH, Coats AJS, Filippatos G, Siddiqi TJ, et al. Patiromer for the management of hyperkalemia in heart failure with reduced ejection fraction: the DIAMOND trial. Eur Heart J. 2022;43(41):4362-73.

11.	Chen HH, Anstrom KJ, Givertz MM, Stevenson LW, Semigran MJ, Goldsmith SR, et al. Low-dose dopamine or low-dose nesiritide in acute heart failure with renal dysfunction: the ROSE acute heart failure randomized trial. JAMA. 2013;310(23):2533-43.

12.	Chung ES, Packer M, Lo KH, Fasanmade AA, Willerson JT. Randomized, double-blind, placebo-controlled, pilot trial of infliximab, a chimeric monoclonal antibody to tumor necrosis factor-alpha, in patients with moderate-to-severe heart failure: results of the anti-TNF Therapy Against Congestive Heart Failure (AT. Circulation. 2003;107(25):3133-40.

13.	Cleland JGF, Kalra PA, Pellicori P, Graham FJ, Foley PWX, Squire IB, et al. Intravenous iron for heart failure, iron deficiency definitions, and clinical response: the IRONMAN trial. Eur Heart J. 2024.

14.	Cohn JN, Johnson G, Ziesche S, Cobb F, Francis G, Tristani F, et al. A comparison of enalapril with hydralazine-isosorbide dinitrate in the treatment of chronic congestive heart failure. N Engl J Med. 1991;325(5):303-10.

15.	Cowley AJ, McEntegart DJ, Hampton JR, Barnett DB, Bexton RS, Boyle R, et al. Long-term evaluation of treatment for chronic heart failure: a 1 year comparative trial of flosequinan and captopril. Cardiovasc Drugs Ther. 1994;8(6):829-36.

16.	Cuffe MS, Califf RM, Adams KF, Jr., Benza R, Bourge R, Colucci WS, et al. Short-term intravenous milrinone for acute exacerbation of chronic heart failure: a randomized controlled trial. JAMA. 2002;287(12):1541-7.

17.	Davies RF, Beanlands DS, Nadeau C, Phaneuf D, Morris A, Arnold JM, et al. Enalapril versus digoxin in patients with congestive heart failure: a multicenter study. Canadian Enalapril Versus Digoxin Study Group. J Am Coll Cardiol. 1991;18(7):1602-9.

18.	DiBianco R, Shabetai R, Kostuk W, Moran J, Schlant RC, Wright R. A comparison of oral milrinone, digoxin, and their combination in the treatment of patients with chronic heart failure. N Engl J Med. 1989;320(11):677-83.

19.	Erdmann E, Semigran MJ, Nieminen MS, Gheorghiade M, Agrawal R, Mitrovic V, et al. Cinaciguat, a soluble guanylate cyclase activator, unloads the heart but also causes hypotension in acute decompensated heart failure. Eur Heart J. 2013;34(1):57-67.

20.	Feldman AM, Oren RM, Abraham WT, Boehmer JP, Carson PE, Eichhorn E, et al. Low-dose oral enoximone enhances the ability to wean patients with ultra-advanced heart failure from intravenous inotropic support: results of the oral enoximone in intravenous inotrope-dependent subjects trial. Am Heart J. 2007;154(5):861-9.

21.	Ghose JC, Chakraborty S, Mondal M, Bhandari B. Effect of vasodilator therapy on mortality in chronic congestive heart failure. J Assoc Physicians India. 1993;41(5):269-71.

22.	Granger CB, Ertl G, Kuch J, Maggioni AP, McMurray J, Rouleau JL, et al. Randomized trial of candesartan cilexetil in the treatment of patients with congestive heart failure and a history of intolerance to angiotensin-converting enzyme inhibitors. Am Heart J. 2000;139(4):609-17.

23.	Granger CB, McMurray JJ, Yusuf S, Held P, Michelson EL, Olofsson B, et al. Effects of candesartan in patients with chronic heart failure and reduced left-ventricular systolic function intolerant to angiotensin-converting-enzyme inhibitors: the CHARM-Alternative trial. Lancet. 2003;362(9386):772-6.

24.	Greenberg B, Butler J, Felker GM, Ponikowski P, Voors AA, Desai AS, et al. Calcium upregulation by percutaneous administration of gene therapy in patients with cardiac disease (CUPID 2): A randomised, multinational, double-blind, placebo-controlled, phase 2b trial. Lancet. 2016;387(10024):1178-86.

25.	Holubarsch CJF, Kohler S, Malek FA, Gaus W. Safe add-on therapy with hawthorn extract WS® 1442 in NYHA II/III heart failure - results of the SPICE study, the rst mortality and morbidity trial with a herbal drug. Perfusion. 2010;23(3):104-9.

26.	Hori M, Sasayama S, Kitabatake A, Toyo-oka T, Handa S, Yokoyama M, et al. Low-dose carvedilol improves left ventricular function and reduces cardiovascular hospitalization in Japanese patients with chronic heart failure: the Multicenter Carvedilol Heart Failure Dose Assessment (MUCHA) trial. Am Heart J. 2004;147(2):324-30.

27.	Jespersen CM. The effect of verapamil on major events in patients with impaired cardiac function recovering from acute myocardial infarction. The Danish Study Group on Verapamil in Myocardial Infarction. Eur Heart J. 1993;14(4):540-5.

28.	Jha V, Aymanom CD, Tiwari S. Randomized, Placebo-Controlled Study to Investigate the Effects of Eplerenone in Patients with Heart Failure of Different Etiologies. Intl J Pharm Clin Res. 2022;14(1):289-94.

29.	Just H, Drexler H, Taylor SH, Siegrist J, Schulgen G, Schumacher M. Captopril versus digoxin in patients with coronary artery disease and mild heart failure. A prospective, double-blind, placebo-controlled multicenter study. The CADS Study Group. Herz. 1993;18 Suppl 1:436-43.

30.	Kjekshus J, Swedberg K. Enalapril for congestive heart failure. Am J Cardiol. 1989;63(8):26D-32D.

31.	Kosiborod MN, Abildstrom SZ, Borlaug BA, Butler J, Rasmussen S, Davies M, et al. Semaglutide in Patients with Heart Failure with Preserved Ejection Fraction and Obesity. N Engl J Med. 2023;389(12):1069-84.

32.	Kosiborod MN, Angermann CE, Collins SP, Teerlink JR, Ponikowski P, Biegus J, et al. Effects of Empagliflozin on Symptoms, Physical Limitations, and Quality of Life in Patients Hospitalized for Acute Heart Failure: Results From the EMPULSE Trial. Circulation. 2022;146(4):279-88.

33.	Kubo SH, Gollub S, Bourge R, Rahko P, Cobb F, Jessup M, et al. Beneficial effects of pimobendan on exercise tolerance and quality of life in patients with heart failure. Results of a multicenter trial. The Pimobendan Multicenter Research Group. Circulation. 1992;85(3):942-9.

34.	Ledwidge M, Dodd JD, Ryan F, Sweeney C, McDonald K, Fox R, et al. Effect of Sacubitril/Valsartan vs Valsartan on Left Atrial Volume in Patients With Pre-Heart Failure With Preserved Ejection Fraction: The PARABLE Randomized Clinical Trial. JAMA Cardiol. 2023;8(4):366-75.

35.	Mann DL, Givertz MM, Vader JM, Starling RC, Shah P, McNulty SE, et al. Effect of Treatment With Sacubitril/Valsartan in Patients With Advanced Heart Failure and Reduced Ejection Fraction: A Randomized Clinical Trial. JAMA Cardiol. 2022;7(1):17-25.

36.	Margulies KB, Hernandez AF, Redfield MM, Givertz MM, Oliveira GH, Cole R, et al. Effects of liraglutide on clinical stability among patients with advanced heart failure and reduced ejection fraction: A randomized clinical trial. JAMA. 2016;316(5):500-8.

37.	Massie BM, Fisher SG, Radford M, Deedwania PC, Singh BN, Fletcher RD, et al. Effect of amiodarone on clinical status and left ventricular function in patients with congestive heart failure. CHF-STAT Investigators. Circulation. 1996;93(12):2128-34.

38.	Massie BM, O'Connor CM, Metra M, Ponikowski P, Teerlink JR, Cotter G, et al. Rolofylline, an adenosine A1-receptor antagonist, in acute heart failure. N Engl J Med. 2010;363(15):1419-28.

39.	McMurray JJ, Ostergren J, Swedberg K, Granger CB, Held P, Michelson EL, et al. Effects of candesartan in patients with chronic heart failure and reduced left-ventricular systolic function taking angiotensin-converting-enzyme inhibitors: the CHARM-Added trial. Lancet. 2003;362(9386):767-71.

40.	Mentz RJ, Garg J, Rockhold FW, Butler J, De Pasquale CG, Ezekowitz JA, et al. Ferric Carboxymaltose in Heart Failure with Iron Deficiency. N Engl J Med. 2023;389(11):975-86.

41.	Mentz RJ, Ward JH, Hernandez AF, Lepage S, Morrow DA, Sarwat S, et al. Angiotensin-Neprilysin Inhibition in Patients With Mildly Reduced or Preserved Ejection Fraction and Worsening Heart Failure. J Am Coll Cardiol. 2023;82(1):1-12.

42.	Morisco C, Trimarco B, Condorelli M. Effect of coenzyme Q10 therapy in patients with congestive heart failure: a long-term multicenter randomized study. Clin Investig. 1993;71(8 Suppl):S134-6.

43.	Mortensen SA, Kumar A, Dolliner P, Filipiak KJ, Pella D, Alehagen U, et al. The effect of coenzyme Q10 on morbidity and mortality in chronic heart failure. Results from the Q-SYMBIO study. European journal of heart failure. 2013;12:S21.

44.	Mortensen SA, Kumar A, Dolliner P, K JF, Pella D, Alehagen U, et al. Long-term result s with coenzyme Q10 as adjunctive therapy in chronic heart failure. Heart (British Cardiac Society). 2013;99:A55-A6.

45.	Mortensen SA, Rosenfeldt F, Kumar A, Dolliner P, Filipiak KJ, Pella D, et al. Coenzyme Q10 therapy blocks the vicious metabolic cycle in chronic heart failure. Journal of cardiac failure. 2013;19(8):S25.

46.	Nieminen MS, Cleland JG, Eha J, Belenkov Y, Kivikko M, Poder P, et al. Oral levosimendan in patients with severe chronic heart failure --the PERSIST study. Eur J Heart Fail. 2008;10(12):1246-54.

47.	Packer M, Butler J, Zannad F, Filippatos G, Ferreira JP, Pocock SJ, et al. Effect of Empagliflozin on Worsening Heart Failure Events in Patients With Heart Failure and Preserved Ejection Fraction: EMPEROR-Preserved Trial. Circulation. 2021;144(16):1284-94.

48.	Packer M, Colucci WS, Sackner-Bernstein JD, Liang CS, Goldscher DA, Freeman I, et al. Double-blind, placebo-controlled study of the effects of carvedilol in patients with moderate to severe heart failure: The PRECISE Trial. CIRCULATION. 1996;94(11):2793-9.

49.	Packer M, McMurray JJ, Desai AS, Gong J, Lefkowitz MP, Rizkala AR, et al. Angiotensin receptor neprilysin inhibition compared with enalapril on the risk of clinical progression in surviving patients with heart failure. Circulation. 2015;131(1):54-61.

50.	Pang PS, Butler J, Collins SP, Cotter G, Davison BA, Ezekowitz JA, et al. Biased ligand of the angiotensin II type 1 receptor in patients with acute heart failure: a randomized, double-blind, placebo-controlled, phase IIB, dose ranging trial (BLAST-AHF). Eur Heart J. 2017;38(30):2364-73.

51.	Petrie MC, Verma S, Docherty KF, Inzucchi SE, Anand I, Belohlavek J, et al. Effect of Dapagliflozin on Worsening Heart Failure and Cardiovascular Death in Patients With Heart Failure With and Without Diabetes. JAMA. 2020;323(14):1353-68.

52.	Pfeffer MA, Swedberg K, Granger CB, Held P, McMurray JJ, Michelson EL, et al. Effects of candesartan on mortality and morbidity in patients with chronic heart failure: the CHARM-Overall programme. Lancet. 2003;362(9386):759-66.

53.	Pratt CM, Singh SN, Al-Khalidi HR, Brum JM, Holroyde MJ, Marcello SR, et al. The efficacy of azimilide in the treatment of atrial fibrillation in the presence of left ventricular systolic dysfunction: results from the Azimilide Postinfarct Survival Evaluation (ALIVE) trial. J Am Coll Cardiol. 2004;43(7):1211-6.

54.	Remme WJ, Krayenbuhl HP, Baumann G, Frick MH, Haehl M, Nehmiz G, et al. Long-term efficacy and safety of pimobendan in moderate heart failure. A double-blind parallel 6-month comparison with enalapril. The Pimobendan-Enalapril Study Group. Eur Heart J. 1994;15(7):947-56.

55.	Rouleau JL, Pfeffer MA, Stewart DJ, Isaac D, Sestier F, Kerut EK, et al. Comparison of vasopeptidase inhibitor, omapatrilat, and lisinopril on exercise tolerance and morbidity in patients with heart failure: IMPRESS randomised trial. Lancet. 2000;356(9230):615-20.

56.	Suzuki S, Yoshihisa A, Yamaki T, Sugimoto K, Kunii H, Nakazato K, et al. Long-term effects and prognosis in acute heart failure treated with tolvaptan: the AVCMA trial. Biomed Res Int. 2014;2014:704289.

57.	Swedberg K, Kjekshus J. Effects of enalapril on mortality in severe congestive heart failure: results of the Cooperative North Scandinavian Enalapril Survival Study (CONSENSUS). Am J Cardiol. 1988;62(2):60A-6A.

58.	Szarek M, Bhatt DL, Steg PG, Cannon CP, Leiter LA, McGuire DK, et al. Effect of Sotagliflozin on Total Hospitalizations in Patients With Type 2 Diabetes and Worsening Heart Failure : A Randomized Trial. Ann Intern Med. 2021;174(8):1065-72.

59.	Taylor AL, Ziesche S, Yancy C, Carson P, D'Agostino R, Jr., Ferdinand K, et al. Combination of isosorbide dinitrate and hydralazine in blacks with heart failure. N Engl J Med. 2004;351(20):2049-57.

60.	Teerlink JR, Metra M, Felker GM, Ponikowski P, Voors AA, Weatherley BD, et al. Relaxin for the treatment of patients with acute heart failure (Pre-RELAX-AHF): a multicentre, randomised, placebo-controlled, parallel-group, dose-finding phase IIb study. Lancet. 2009;373(9673):1429-39.

61.	Torp-Pedersen C, Kober L, Carlsen J. Angiotensin-converting enzyme inhibition after myocardial infarction: the Trandolapril Cardiac Evaluation Study. Am Heart J. 1996;132(1 Pt 2 Su):235-43.

62.	Tsutsui H, Momomura SI, Masuyama T, Saito Y, Komuro I, Murohara T, et al. Tolerability, Efficacy, and Safety of Bisoprolol vs. Carvedilol in Japanese Patients With Heart Failure and Reduced Ejection Fraction - The CIBIS-J Trial. Circ J. 2019;83(6):1269-77.

63.	Udelson JE, McGrew FA, Flores E, Ibrahim H, Katz S, Koshkarian G, et al. Multicenter, randomized, double-blind, placebo-controlled study on the effect of oral tolvaptan on left ventricular dilation and function in patients with heart failure and systolic dysfunction. J Am Coll Cardiol. 2007;49(22):2151-9.

64.	Vizzardi E, Nodari S, Caretta G, D'Aloia A, Pezzali N, Faden G, et al. Effects of spironolactone on long-term mortality and morbidity in patients with heart failure and mild or no symptoms. Am J Med Sci. 2014;347(4):271-6.

65.	Voors AA, Bax JJ, Hernandez AF, Wirtz AB, Pap AF, Ferreira AC, et al. Safety and efficacy of the partial adenosine A1 receptor agonist neladenoson bialanate in patients with chronic heart failure with reduced ejection fraction: a phase IIb, randomized, double-blind, placebo-controlled trial. Eur J Heart Fail. 2019;21(11):1426-33.

66.	Win S, Anand I, Rector T, Furst H, Cohn J, Taylor AL. Combination of isosorbide dinitrate and hydralazine reduces 30 day hospital readmissions and increases time to hospital readmission in blacks with heart failure. Journal of the American College of Cardiology. 2012;59(13):E1042.

67.	Yang W, Zhou YJ, Fu Y, Qin J, Qin S, Chen XM, et al. Efficacy and Safety of Intravenous Urapidil for Older Hypertensive Patients with Acute Heart Failure: A Multicenter Randomized Controlled Trial. Yonsei Med J. 2017;58(1):105-13.

68.	Yusuf S. Reduced mortality and morbidity with the use of angiotensin-converting enzyme inhibitors in patients with left ventricular dysfunction and congestive heart failure. Herz. 1993;18 Suppl 1:444-8.

69.	Yusuf S, Pepine CJ, Garces C, Pouleur H, Salem D, Kostis J, et al. Effect of enalapril on myocardial infarction and unstable angina in patients with low ejection fractions. Lancet. 1992;340(8829):1173-8.

70.	Yusuf S, Teo K, Anderson C, Pogue J, Dyal L, Copland I, et al. Effects of the angiotensin-receptor blocker telmisartan on cardiovascular events in high-risk patients intolerant to angiotensin-converting enzyme inhibitors: a randomised controlled trial. Lancet. 2008;372(9644):1174-83.

71.	Zeng XH, Zeng XJ, Li YY. Efficacy and safety of berberine for congestive heart failure secondary to ischemic or idiopathic dilated cardiomyopathy. Am J Cardiol. 2003;92(2):173-6.

72.	Zhao Y, Krupadev V, Dagher L, Mahnkopf C, Sohns C, Sehner S, et al. Pharmacological rhythm versus rate control in patients with atrial fibrillation and heart failure: the CASTLE-AF trial. J Interv Card Electrophysiol. 2021;61(3):609-15.

73.	Yang DY, P.; Li, X.; Liu, Y.; You, H.; Lui, P. . Efficacy and Safety of Omecamtiv Mecarbil in Adult Patients with Heart Failure with Reduced Ejection Fraction. International Journal of Pharmacology. 2024;20:964-72.

74.	Shabana Mohammad A. Safety of Dapagliflozinin Reducing Cardiac Events and Deaths among NYHA Class II and III Cardiac Failure Patients. Pakistan Journal of Medical & Health Sciences. 2024;18(01):35.
